# The application of straw returning combined with low-temperature degrading microbial inoculant M44 in cold and arid regions promotes the efficient decomposition of returned straw through the hierarchical interaction mechanism of “key microorganisms—bacterial community structure—extracellular enzyme activity—straw degradation”

**DOI:** 10.3389/fmicb.2026.1765717

**Published:** 2026-04-29

**Authors:** Wenshan Zhao, Lijie Wang, Ruizhi Liu, Haoran Jiang, Dongbo Li, Shuping Hu, Julin Gao, Xiaofang Yu, Qinggeer Borjigin

**Affiliations:** 1 Engineering Technology Research Center for Microorganisms of Maize Straw Returning to Field in Situ in Inner Mongolia Autonomous Region, Hohhot, Inner Mongolia, China; 2 Key Laboratory of Crop Cultivation and Genetic Improvement in Inner Mongolia Autonomous Region, Hohhot, Inner Mongolia, China; 3 Inner Mongolia Agricultural University Vocational and Technical College, Baotou, Inner Mongolia, China

**Keywords:** decay-promoting mechanism, extracellular enzyme activity, microbial community, microbial inoculant M44, straw degradation, straw return methods

## Abstract

To address the bottleneck problem of slow decomposition of returned maize straw under low-temperature constraints in the cold and arid regions of northern China, this study systematically explored the microbial decomposition-promoting mechanism of microbial inoculant M44 combined with three straw returning methods: deep plowing (DPR), deep scarification and mixing (SSR), and no-tillage mulching (NTR), by integrating field tillage and in-situ micro-zone degradation experiments. The results showed that different straw returning methods combined with inoculant M44 could effectively overcome low-temperature limitations, significantly increasing the straw degradation rate by 2.33–9.81 percentage points and shortening the half-life by 20.7–62.8 d. The core mechanism was that the “tillage-inoculant” interaction regulated the soil microenvironment, directionally shaped and enriched key functional microbial taxa with degradation ability (such as *Pseudoxanthomonas*, *Devosia*, *Streptomyces*, *Pseudomonas*, etc.) and their key ASVs (such as ASV6, ASV12, ASV412, ASV1546, etc.), reshaped the soil bacterial community structure, and synergistically activated the soil extracellular enzyme system (such as β-glucosidase, β-xylosidase, etc., with the comprehensive enzyme index increased by 0.74–1.06), thereby synergistically driving the rapid degradation of returned straw. The PLS-PM model further clarified that there were differences in the pathways driven by inoculant M44 for straw degradation under different returning methods. In the DPR and SSR treatments, bacterial community composition was the most important direct driving force for degradation, and key ASVs indirectly affected the degradation process by regulating bacterial composition and enzyme activity, while in the NTR treatment, extracellular enzyme activity became the core driving force for degradation, whose activity was directly driven by bacterial composition and diversity. This study revealed the hierarchical interaction driving mechanism of “key microorganisms—bacterial community structure—extracellular enzyme activity—straw degradation” at the field scale, providing an important scientific basis for optimizing the “tillage-inoculant” synergistic technology for straw resource utilization in cold and arid regions.

## 1 Introduction

Straw returning is a key practice for enhancing soil fertility, improving soil structure, and promoting sustainable agricultural development. It is particularly critical in arid and semi-arid regions for strengthening soil water and nutrient retention capacity (Zhao et al., 2021; Ding et al., 2025). However, in cold and arid northern regions, the low temperatures and limited rainfall during autumn, winter, and early spring significantly inhibit microbial activity, leading to slow decomposition of returned maize straw. This not only affects soil tilth and hinders the expansion of soil carbon sinks and the improvement of nutrient cycling efficiency, but also severely constrains maize sowing and seedling emergence (Zhang et al., 2023; Ji et al., 2024). Maize straw is primarily composed of lignocellulose, it’s complex chemical composition and compact spatial structure make it resistant to efficient decomposition under natural conditions (Bature et al., 2025). To address this bottleneck, the development and application of microbial agents capable of functioning under low-temperature conditions have emerged as a critical strategy for enhancing the decomposition of returned maize stover (Yao et al., 2023).

The degradation of straw requires the synergistic action of various hydrolytic enzymes, primarily including β-glucosidase, β-cellobiosidase, β-xylosidase, and laccase (Gharechahi et al., 2023). During the straw degradation process, key microbial genera such as *Bacillus*, *Pseudomonas*, *Clostridium*, *Streptomyces*, and *Cellulomonas* secrete these relevant hydrolytic enzymes, thereby facilitating straw degradation (Cruz et al., 2024). Therefore, previous studies have focused on screening and applying single or composite microbial strains for straw degradation. For instance, Gong et al. (2020) constructed an in-situ degradation microbial consortium by combining three Streptomyces strains (G1, G2, and G3). Under optimized conditions (pH 7, temperature 30 °C, inoculation amount 5%, rotation speed 210 rpm), the straw weight loss rate reached 60.55% after 7 days of degradation. Chen et al. (2021) isolated and identified a cellulase-producing bacterium (*Acetobacter orientalis* XJC-C) from marine soft corals, which exhibited salt and high-temperature tolerance. The lignin degradation rate in the treatment group reached 76.24%, representing increases of 47.08 and 21.85% compared to the without bacterial inoculation and inoculated with a standard cellulose-decomposing strain, respectively. He et al. (2023) screened a low-temperature efficient cellulose-degrading strain, *Bacillus subtilis* K1, from maize straw compost at an ambient temperature of 6 °C. The lignocellulose degradation rate increased by 18.01–41.39% compared to the non-inoculated control group. Wei et al. (2018) demonstrated that inoculating thermophilic actinomycetes during composting not only altered the structure of actinobacterial and bacterial communities but also accelerated the degradation of cellulose, hemicellulose, and lignin, while enhancing the activity of key enzymes such as CMCase, xylanase, manganese peroxidase, lignin peroxidase, and peroxidase. Wang et al. (2023) investigated the effects of adding an exogenous lignocellulose-degrading microbial consortium on rice straw return at different soil depths in cold regions. They found that the composite microbial consortium also modified the native soil microbial community structure, effectively increased the functional richness of soil microorganisms, and identified dominant strains such as SJA-15, *Gemmatimonadaceae*, and *Bradyrhizobium* that promoted straw degradation.

In a previous study, our laboratory successfully screened a straw-degrading microbial consortium M44 through low-temperature restricted subcultivation. This consortium demonstrated stable and efficient straw degradation at temperatures of 10–15 °C and pH 6–8. The maize straw degradation rate was 12.25% higher than that of the control consortium (GF-20). Additionally, the activities of filter paper enzyme, endoglucanase, xylanase, and laccase were 7.41, 1.44, 13.85, and 17.88% higher, respectively, compared to the control (Zhang et al., 2021a,b). Field experiments further demonstrated that by the end of the decomposition period, the degradation rates of maize straw, cellulose, hemicellulose, and lignin under the straw-degrading bacterial treatment reached 58.13–62.53%, 39.38–47.71%, 39.91–49.96%, and 30.97–38.79%, respectively (Zhang, 2022), indicating good application potential. However, it should be noted that different straw return methods significantly affect microbial colonization efficiency and the straw degradation process by creating distinct soil microenvironments (Min et al., 2019). Current research on straw-degrading bacteria primarily focuses on evaluating the decay-promoting effects of microbial inoculants (Niu et al., 2025; Zhang M. et al., 2025; Shamshitov et al., 2025), but lacks systematic analysis of the “tillage-inoculant” interaction mechanisms regulating maize straw degradation pathways under typical straw return modes. Furthermore, the impact of microbial agents on the structural diversity and network stability of indigenous soil microbial communities, as well as extracellular enzyme activities in complex field environments, requires further validation. The interactions between inoculants and indigenous microorganisms, as well as their mechanisms in soil microecological reconstruction, have not yet been elucidated.

This study integrated field tillage practices with an improved in-situ nylon mesh bag micro-plot degradation assay to systematically investigate in the sandy loam spring maize cultivation area of Tumochuan Plain, Inner Mongolia. The research systematically investigated: (1) the dynamics patterns of maize straw degradation and lignocellulose decomposition rate following the application of microbial inoculant M44 under three tillage methods: deep plowing with straw return (DPR), deep loosening and mixing with straw return (SSR), and no-tillage with straw mulching (NTR); (2) the regulatory mechanisms of the tillage-inoculant interaction on soil extracellular enzyme activities, microbial community structure, and network stability; and (3) through interaction correlation analysis of tillage and inoculant, to elucidate the microbial driving mechanisms of maize straw degradation under the three-tier interaction of “key microorganisms-extracellular enzyme activity-straw degradation,” thereby revealing the decay-promoting mechanisms of straw under different straw return methods combined with M44 application. The findings provide scientific evidence for optimizing synergistic “tillage-microbial agent” technologies in cold, arid and semi-arid regions, thus actively promoting efficient utilization of straw resources and sustainable soil development.

## 2 Materials and methods

### 2.1 Overview of the experiment

The experiment was conducted at the China Chilechuan Modern Agricultural Expo Garden experimental site (40°32′N, 110°28′E) in Tumed Right Banner, Baotou City, Inner Mongolia Autonomous Region. Located in the Tumochuan Plain, the area experiences a semi-arid mid-temperate continental monsoon climate. The soil type is sandy loam, with spring maize as the previous crop. After maize harvest, the straw was directly crushed and returned to the field. Prior to the experiment, the soil in the 0–45 cm layer of the experimental area contained 21.84 g/kg organic matter, 64.51 mg/kg alkali-hydrolyzable nitrogen, 10.08 mg/kg available phosphorus, and 88.68 mg/kg available potassium. The test maize straw was obtained from harvested in the same experimental field, with the variety Xianyu 696 (35.10% cellulose, 29.40% hemicellulose, and 15.10% lignin). The dominant genera in the straw-degrading composite inoculant M44 (It was stored in the microbial strain resource bank of Maize Center, Inner Mongolia Agricultural University, numbered IMAU-MCGFM44) were *Pseudomonas*, *Devosia*, *Azospirillum*, *Trichococcus*, *Acinetobacter*, *Rhizobium*, *Achromobacter*, *Brevundimonas*, *Chryseobacterium*, *Flavobacterium*, and *Dysgonomonas*, with a total effective viable count of ≥1.0 × 10^9^ CFU/g (Zhang et al., 2022). The average daily soil temperature and humidity during the experimental period are provided in the Supplementary Figure S1.

### 2.2 Experimental design

This experiment employed a split-plot design with three main-plot treatments: deep plow straw return (DPR), deep loosening with mixing straw return (SSR), and no-till with mulching straw return (NTR). The sub-plot treatments consisted of microbial agent application (M44) and a non-application control (CK). Each treatment covered an area of 200 m^2^ (containing five replicate plots per treatment), with buffer zones of equal area separating adjacent treatments to prevent cross-contamination. After maize harvest, the straw underwent secondary crushing. Prior to tillage treatments, urea was broadcast at a rate of 150 kg/ha. On the same day, the dry powder microbial agent M44 was uniformly mixed with appropriate amount of soil from the same field at suitable moisture content and applied to the straw surface at a rate of 30 kg/ha. All operations for the control treatment remained identical, except for the omission of the microbial agent.

Simultaneously, an in-situ microplot degradation experiment was conducted using an improved nylon mesh bag method. Thirty nylon mesh bags (15 cm × 25 cm, 0.15 mm aperture) were uniformly distributed in each plot of every treatment, totaling 180 bags. Each bag contained 20 g of maize straw that had been washed, oven-dried at 60 °C to constant weight, and cut into 3–5 cm segments, along with 1 kg of soil from the same tillage layer and 0.04 g of dry powder inoculant M44. The control treatments underwent identical procedures except for the omission of the inoculant. The burial methods varied by treatment: Deep plow straw return (DPR): Bags were buried at 35–40 cm depth and backfilled with compacted soil. Deep loosening with mixing straw return (SSR): Bags were vertically buried at 10–30 cm depth and backfilled with compacted soil. No-till with mulching straw return (NTR): Bags were horizontally placed on the soil surface at 0–5 cm depth. The two-year repeated experiment initiated bag burial on November 9, 2022, and November 7, 2023. Six bags were retrieved from each plot at 140, 185, 230, 275, and 320 days after burial. Retrieved straw was carefully separated, washed, dried at 60 °C to constant weight, and weighed to calculate degradation rates before being ground for lignocellulose degradation analysis. Surrounding soil from each bag was divided into two uniform portions after removing plant and animal residues: One part was stored at 4 °C and −86 °C for soil extracellular enzyme activity analysis, while the other was air-dried at room temperature. The straw-adhering soil (soil attached to the straw surface) was stored at −86 °C for analysis of soil microbial community structural diversity.

### 2.3 Measurement indicators and methods

#### 2.3.1 Determination of straw degradation indexes

The maize straw degradation rate was determined using the weight loss method. The contents of cellulose, hemicellulose, and lignin in straw were determined using a fiber analyzer (ANKOM DELTA, United States). The percentage contents of neutral detergent fiber (NDF), acid detergent fiber (ADF), acid detergent lignin (ADL), and silicate were determined, and the lignocellulose content was subsequently calculated. The apparent structure of straw was examined using a scanning electron microscope (TM4000 Plus). SEM images were acquired at an accelerating voltage of 5 kV and a magnification of 400×. The relationship between straw residual rate and degradation time was fitted using the first-order decay exponential equation: *y_t_* = *y*_0_ + α × *e*^(−kt)^. Where: *y_t_* represents the mass residual rate (%) of maize straw at time *t*; *y*_0_ denotes the asymptotic residual rate when *t* approaches infinity; α indicates the proportion of degradable straw component; and *k* is the decay rate constant. The decomposition rate constant was calculated as *K* = −ln(*X_t_*/*X*_0_)/*t*, where *X_t_* is the residual mass of straw at time *t*, *X*_0_ is the initial mass of straw, and *t* is the degradation time. The staged degradation rate of straw was calculated as DD (%) = (*D_n_* - *D_m_*)/*t*(*n*-*m*), where n and m represent different degradation time points, *D_n_* is the straw degradation rate at time *n*, and *D_m_* is the straw degradation rate at time m.

#### 2.3.2 Determination of extracellular enzyme activities

The activities of β-glucosidase, β-cellobiosidase, β-xylosidase, laccase, N-acetyl-β-glucosaminidase, and leucine aminopeptidase were measured using a microplate fluorometer. The soil extracellular enzyme composite index (EMF) serves as a metric for assessing soil ecosystem multifunctionality based on enzyme activities. Each soil enzyme activity was standardized using the Z-score method, and the mean value was then calculated. The calculations were performed as follows: *Z_i_* = (*x* - *m_i_*) / sd; Z-score = average (*Z_i_*); where *Z_i_* represents the standardized enzyme activity, *x* is the measured enzyme activity, m_i_ is the mean enzyme activity, and sd is the standard deviation.

#### 2.3.3 Analysis of microbial diversity

The microbial community determination focused on bacterial data, and the samples used were collected in 2023. All soil samples were collected following the standard sampling protocol provided by Majorbio Bio-pharm Technology Co., Ltd. They were immediately transported to the laboratory on dry ice to maintain the stability of the bacterial community and stored at −80 °C for subsequent DNA extraction.

1. DNA extraction

Total bacterial genomic DNA was extracted from soil samples using the FastPure® Soil DNA Isolation Kit (MJYH, Shanghai, China) strictly following the manufacturer’s instructions. DNA quality was assessed by 1.0% agarose gel electrophoresis, and the concentration and purity of DNA were determined using a NanoDrop 2000 spectrophotometer (Thermo Scientific, United States). The extracted bacterial DNA was stored at −80 °C until subsequent PCR amplification to avoid degradation.

1. PCR amplification

The hypervariable V3-V4 region of the bacterial 16S rRNA gene was amplified using specific primer pairs: forward primer 338F (5′-ACTCCTACGGGAGGCAGCAG-3′) and reverse primer 806R (5′-GGACTACHVGGGTWTCTAAT-3′).

The PCR reaction was performed in a 20 μL system containing 10 μL 2 × Pro Taq, 0.8 μL forward primer (5 μM), 0.8 μL reverse primer (5 μM), 10 ng template DNA, and ddH2O to make up the final volume. The amplification was carried out on an ABI GeneAmp® 9700 thermal cycler with the following conditions: initial denaturation at 95 °C for 3 min; 29 cycles of denaturation at 95 °C for 30 s, annealing at 53 °C for 30 s, and extension at 72 °C for 45 s; final extension at 72 °C for 10 min, followed by holding at 10 °C until halted by the user.

PCR products were detected by 2% agarose gel electrophoresis. Target bands with correct size and appropriate concentration (result code “A” as defined in the PCR report) were purified using the PCR Clean-Up Kit (YuHua, Shanghai, China) according to the manufacturer’s instructions.

1. Illumina sequencing and bioinformatics analysis

Purified bacterial amplicons were pooled in equimolar amounts and paired-end sequenced on an Illumina Nextseq2000 platform (Illumina, San Diego, United States) by Majorbio Bio-pharm Technology Co., Ltd. (Shanghai, China) following standard protocols.

Raw reads were quality-filtered using fastp (version 0.19.6) and merged with FLASH (version 1.2.11). High-quality sequences were denoised using the DADA2 plugin in the QIIME2 (version 2024) pipeline to obtain amplicon sequence variants (ASVs) of bacteria. To minimize the impact of sequencing depth on bacterial diversity analysis, all samples were rarefied to 20,000 sequences. The average Good’s coverage of each sample was 97.90%, indicating sufficient sequencing depth to capture the bacterial community diversity.

Taxonomic annotation of bacterial ASVs was performed using the Naive Bayes classifier in QIIME2 against the SILVA 16S rRNA database (v138.2) with a confidence threshold of 70%. Functional prediction of the bacterial community was conducted using PICRUSt2 (version 2.2.0) software to infer potential metabolic pathways related to bacteria.

#### 2.3.4 Screening and identification of key ASVs and bacterial genera

Rarefied raw microbial sequencing data were acquired from the Meiji Cloud Platform. After Illumina sequencing and preliminary bioinformatic processing, we performed the following analytical steps: First, the raw absolute abundance tables were converted to relative abundances to eliminate biases induced by uneven sequencing depth among different samples. The normalized data were then grouped by tillage practice [deep plowing with straw return (DPR), deep loosening and harrowing with straw return (SSR), no-till mulching with straw return (NTR)]. Next, ASVs were ranked according to their cumulative relative abundances across all samples, and the top 1‰ ASVs were selected to reconstruct a streamlined core ASV dataset. This threshold was selected because high-abundance ASVs represent ecologically dominant taxa with stronger functional relevance. The core ASV dataset was then re-uploaded to the Meiji Cloud Platform for the subsequent analyses: 1. Species succession analysis: ASV activity dynamics were tracked across all straw degradation stages to identify active ASVs that maintained high abundance during key degradation periods and exhibited temporal responsiveness (i.e., “active ASVs”). 2. Spearman’s correlation analysis: Spearman’s rank correlation analysis was performed between the relative abundances of core ASVs and functional indicators. A significant correlation was defined as *P* < 0.05 with |Spearman’s correlation coefficient *P*| > 0.5 to ensure the reliability of ecological associations. 4. The core ASV dataset was processed using the psych and vegan packages in R (v4.3.3) to calculate pairwise Spearman’s rank correlations. The resulting correlation matrix was imported into Gephi (v0.10.1) to construct a co-occurrence network: node size represents ASVs and is proportional to their average relative abundance; edges represent significant correlations (|*P*| > 0.6, *P* < 0.01), with colors distinguishing correlation directions (red for positive correlations, blue for negative correlations). Core nodes were defined as those with high topological centrality, which indicates their key role in the ecological network. Finally, the key ASVs were identified by integrating three lines of evidence from the above analyses. Based on the rarefied data from the Meiji Cloud Platform, key bacteria at the genus level were screened using two analytical approaches: The Student’s *t*-test was applied to evaluate abundance differences of bacterial genera among different tillage practices (DPR/SSR/NTR); with *P* < 0.05 set as the significance threshold, the top 15 bacteria that contributed most significantly to intergroup classification were screened at the genus level. Subsequently, a random forest classification model was constructed to ensure that the screened bacterial genera at the genus level exhibited stable taxonomic contributions.

### 2.4 Statistical analysis

Statistical analyses were performed as follows: Data organization and classification were conducted using Microsoft Excel 2019. Significance tests were performed with IBM SPSS Statistics 26.0. Heatmaps based on Spearman’s correlation tests were generated using R-3.3.1 and Python-2.7. Student’s *t*-test was employed to assess significance between M44 inoculant application and non-inoculated treatments. Network diagrams were constructed with Gephi 0.10.1. Random forest modeling was implemented using R-3.3.1 (randomForest package) to evaluate predictor variable importance through assessment of predictive accuracy reduction. Partial least squares path modeling (PLS-PM) was applied to analyze direct and indirect factors affecting straw degradation efficiency, where path coefficients indicate the direction and strength of linear relationships between latent variables, and R^2^ represents the percentage of variance explained. The PLS-PM was constructed using the “plspm” package in R-4.3.3. Other data plotting and equation fitting were performed using Origin 2021.

## 3 Results and analysis

### 3.1 Effects of straw return methods with inoculant application on straw degradation

**Figure 1:**
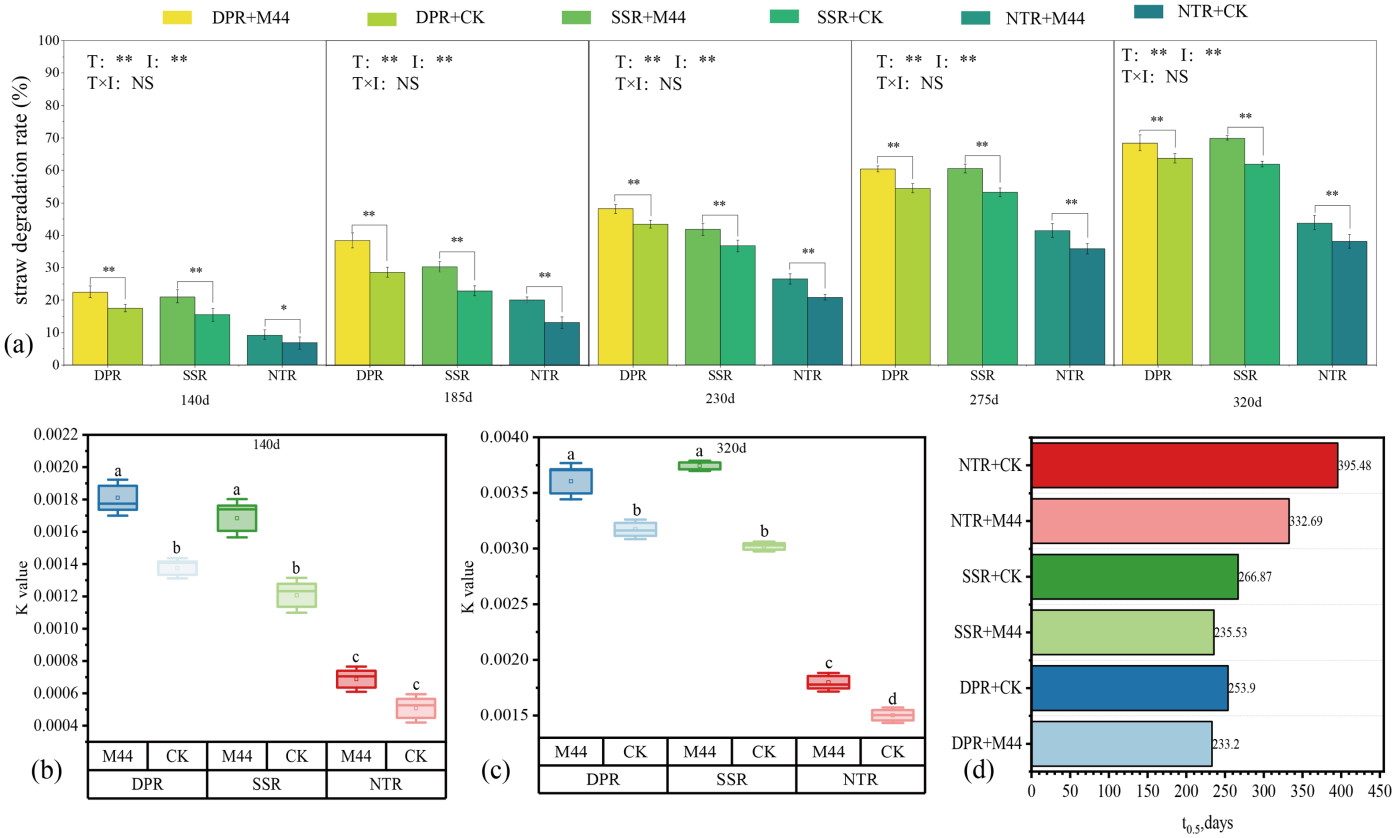
Straw degradation efficiency under different straw return methods with microbial agent application. **(a)** Straw degradation rates across sampling periods under different straw return methods with microbial agent application. T and I represent tillage method and inoculation treatment, respectively; * and ** indicate significant effects at the 0.05 and 0.01 probability levels, respectively; NS indicates non-significant effects. **(b,c)** Decomposition constants at 140 d and 320 d under different straw return methods with microbial agent application. Different lowercase letters indicate significant differences among treatments (*P* < 0.05). **(d)** Half-life (t₀.₅, days) of straw degradation under different straw return methods with microbial agent application.

Significant differences (*P* < 0.05) were observed among tillage methods and inoculant application treatments at each sampling period during the two-year study. In 2023, the straw degradation rates of the DPR + M44, SSR + M44, and NTR + M44 treatments were significantly higher than those of the non-inoculated control by 4.63–9.81, 5.05–7.88, and 2.33–6.98 percentage points, respectively. In 2024, these rates were significantly increased by 2.79–8.20, 3.50–10.14, and 2.79–8.20 percentage points, respectively (*P* < 0.05). Among different tillage practices, the decay-promoting effects of DPR and SSR were significantly superior to those of NTR both in 2023 and 2024, with increases of 10.66–25.60, 8.69–26.08, and 11.11–25.81, 9.90–27.38 percentage points, respectively (*P* < 0.05) (Figure 1a; Supplementary Figure S2a). In 2023, DPR + M44, SSR + M44, and NTR + M44 treatments significantly enhanced straw degradation rates during the 0–185 day period compared to the non-inoculated control, with increases of 4.88–4.93, 5.45, and 2.01–5.45 percentage points, respectively. In 2024, these treatments significantly increased straw degradation rates during the 0–140 day period by 8.20 and 3.76 percentage points, respectively (*P* < 0.05) (Table 1; Supplementary Table S1). Further analysis using the first-order decay exponential equation *yt* = *y*_0_ + α × *e*^(−kt)^ to fit the relationship between straw residual rate and decomposition time revealed that over the 2 years, the decomposition constants *K* of DPR + M44, SSR + M44, and NTR + M44 treatments at 140 and 320 days were significantly higher than those of the non-inoculated control, with increases of 37 × 10^−5^ to 67 × 10^−5^, 48 × 10^−5^ to 129 × 10^−5^, and 18 × 10^−5^ to 65 × 10^−5^, respectively. Among different tillage practices, the decomposition constants K for DPR and SSR were consistently higher than those for NTR, differing by 87 × 10^−5^ to 181 × 10^−5^ and 70 × 10^−5^ to 194 × 10^−5^, respectively (Figures 1b,c; Supplementary Figures S2b,c). The half-life of straw degradation (*t*_0.5_, days) for DPR + M44, SSR + M44, and NTR + M44 treatments was shortened by 20.7–25.8, 31.3–32.2, and 25.4–62.8 days, respectively, compared to the non-inoculated control over the 2 years (Figure 1d; Supplementary Figure S2d). These results demonstrate that application of the composite microbial inoculant M44 significantly enhanced straw degradation rates compared to the non-inoculated control, with more pronounced improvements during autumn and the low-temperature period in spring. The annual straw degradation effect for 2024 is shown in Supplementary Figure S2 and Supplementary Table S1.

**Table 1:** Straw degradation rates at different decomposition stages across treatments (%).

Treatment	Degradation time (d)					Fitted equation	*R*^2^ value
	0–140 d	140–185 d	185–230 d	230–275 d	275–320 d		
DPR+M44	22.38a	16.03a	9.75b	12.26c	7.95a	*y* = −29.34 + 167.33e^−0.0032x^	0.994
DPR−CK	17.50b	11.10b	14.84a	11.04c	9.26a	*y* = −75.40 + 208.37e^−0.0020x^	0.995
SSR+M44	20.98a	9.36bc	11.45b	18.75a	9.27a	*y* = −28.59 + 174.51e^−0.0034x^	0.998
SSR−CK	15.53b	7.35 cd	13.86a	16.54b	8.65a	*y* = −64.89 + 201.22e^−0.0021x^	0.996
NTR+M44	9.17c	10.92b	6.52c	14.77b	2.35b	*y* = −38.84 + 167.65e^−0.0019x^	0.957
NTR−CK	6.84d	6.27d	7.77c	15.02b	2.24b	*y* = −68.50 + 190.50e^−0.0012x^	0.998

Different lowercase letters within the same column indicate significant differences among treatments (*P* < 0.05).

**Figure 2:**
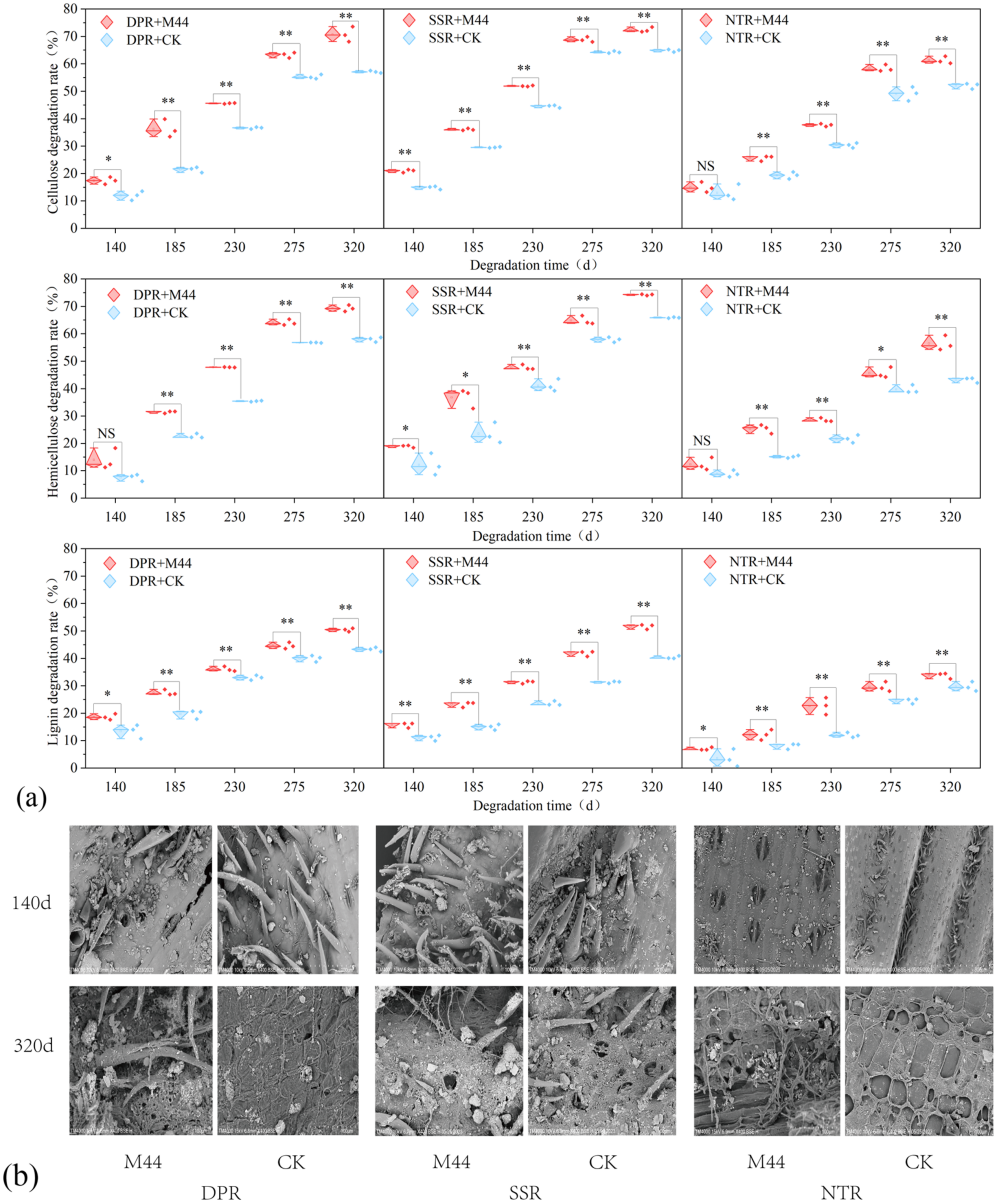
Lignocellulose degradation and apparent structure under different straw return methods. **(a)** Lignocellulose degradation rates across sampling periods. * and ** indicate significance at *P* < 0.05 and *P* < 0.01 levels, respectively; NS indicates non-significant effects. **(b)** Scanning electron microscopy images of straw apparent structures.

Over the two-year study, except for a few treatment groups in certain periods, the degradation rates of cellulose, hemicellulose, and lignin in DPR + M44, SSR + M44, and NTR + M44 treatments were significantly higher than the control at each sampling period. The increases for cellulose were 5.19–14.81, 3.53–10.02, and 4.10–12.41 percentage points, respectively (*P* < 0.05); for hemicellulose, 7.27–24.62, 3.42–13.21, and 3.75–15.84 percentage points (*P* < 0.05); and for lignin, 3.10–8.55, 3.93–12.31, and 3.41–11.18 percentage points (*P* < 0.05). Among different tillage practices, both years demonstrated that DPR and SSR had superior lignocellulose degradation-promoting effects compared to NTR, with increases of 2.11–20.97 and 2.58–26.67 percentage points, respectively (Figure 2a; Supplementary Figure S3). Examination of the apparent structural morphology of straw in 2023 revealed abundant microbial colonization on the surface of straw from DPR + M44, SSR + M44, and NTR + M44 treatments throughout all periods. As degradation progressed, the inoculated treatments exhibited unevenly distributed pores and exposed fibrous structures. In contrast, only minor and fine pores were visible on the straw surfaces in the non-inoculated treatments, with no significant microbial colonization evident (Figure 2b). These findings demonstrate that the application of the straw-decomposing microbial agent M44 effectively promotes the structural breakdown of maize straw tissues and facilitates lignocellulose degradationThe lignocellulose degradation rates of straw with microbial agent application under different straw return methods in 2024 are shown in Supplementary Figure S3.

### 3.2 Effects of straw return methods with inoculant application on soil extracellular enzyme activities

**Figure 3:**
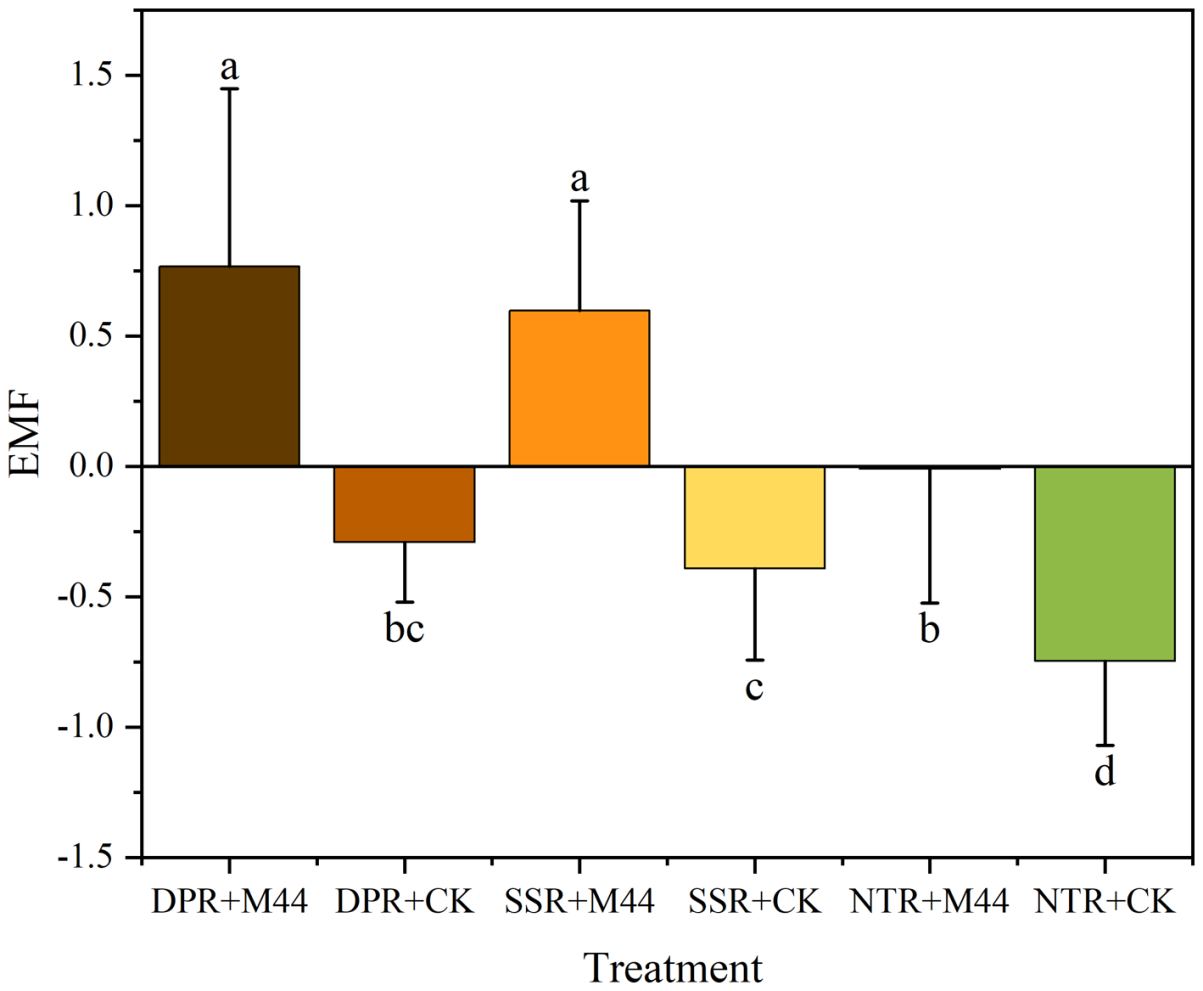
Comprehensive enzyme index of different straw returning methods combined with inoculant application. Different lowercase letters indicate significant differences between treatments (*P* < 0.05).

At all sampling periods in 2023, the combination of different straw return methods with microbial agent application significantly enhanced soil extracellular enzyme activities (Table 2). Specifically, compared with non-inoculated treatments, the inoculated treatments showed increases in β-xylosidase, β-glucosidase, β-cellobiosidase, N-acetylglucosaminidase, and laccase activities by 2.25–22.89, 3.43–25.02, 2.74–26.74, 0.92–5.32, and 0.55–18.39 nmol/(g·h), respectively (*P* < 0.05), with the exception of β-cellobiosidase and laccase at 320 d. Leucine aminopeptidase activity in the inoculated treatments was significantly higher than that in non-inoculated treatments only at 0–185 d, with an increase of 2.58–5.60 nmol/(g·h) (*P* < 0.05) (Figure 3a). The comprehensive enzyme activity index in DPR + M44, SSR + M44, and NTR + M44 treatments was increased by 1.06, 0.99, and 0.74, respectively, compared with non-inoculated treatments (*P* < 0.05). Furthermore, the comprehensive enzyme activity indices in DPR and SSR treatments were superior to those in NTR, with increases of 0.46–0.78 and 0.35–0.61, respectively (Figure 3). These results demonstrate that the application of the composite microbial agent M44 significantly enhanced soil extracellular enzyme activities to varying degrees, and the soil extracellular enzyme activities exhibited differential responses to the combination of different straw return methods with microbial agent application, with the overall performance in the order of DPR > SSR > NTR.

**Table 2:** Changes in extracellular enzyme activities in soil during different degradation stages of various treatments with microbial inoculant in 2023 (nmol/(g·h)).

Degradation time	Treatment	β-Xylosidase	β-Glucosidase	β-Cellobiosidase	L-Leucine aminope-ptidase	N-Acetyl glucosami-dase	Laccase
140 d	DPR + M44	17.3 ± 1.04a	39.02 ± 2.95a	103.28 ± 2.16a	31.74 ± 0.68a	16.99 ± 0.61a	44.96 ± 0.93a
	DPR + CK	5.97 ± 0.63c	19.06 ± 1.41c	79.79 ± 2.69d	28.3 ± 0.39c	15.4 ± 0.3b	26.58 ± 0.53e
	SSR + M44	15.49 ± 0.27b	36.78 ± 0.68a	99.23 ± 0.84a	29.62 ± 0.59b	15.58 ± 0.61b	39.26 ± 1.47b
	SSR + CK	5.53 ± 0.74c	18.19 ± 2.88c	83.87 ± 1.7c	26.26 ± 0.29d	13.8 ± 0.61c	27.16 ± 0.21e
	NTR + M44	14.31 ± 0.22b	32.12 ± 0.96b	93.63 ± 0.4b	26.24 ± 0.36d	13.48 ± 0.79 cd	36.45 ± 1.13c
	NTR + CK	4.84 ± 1.38c	13.19 ± 1.27d	86.05 ± 3.97c	23.66 ± 0.78e	12.57 ± 0.63d	29.29 ± 0.38d
185 d	DPR + M44	21.05 ± 0.73a	41.03 ± 1.85a	108.66 ± 0.51a	34.65 ± 0.31a	25.2 ± 0.77a	28.3 ± 0.78a
	DPR + CK	9.94 ± 0.42c	21.9 ± 1.13c	81.92 ± 0.54e	29.04 ± 0.32c	23.31 ± 0.89b	24.82 ± 1.63bc
	SSR + M44	20.29 ± 0.35a	37.84 ± 0.5ab	108.25 ± 1.25a	33.15 ± 0.11b	19.45 ± 0.69c	26.37 ± 0.22b
	SSR + CK	7.65 ± 0.72d	19.17 ± 1.1 cd	88.83 ± 1.02d	28.13 ± 0.78d	15.9 ± 0.79e	23.39 ± 0.99 cd
	NTR + M44	15.07 ± 0.53b	35.22 ± 0.69b	97.37 ± 3.34b	28.52 ± 0.45 cd	18.04 ± 0.53d	24.17 ± 0.21c
	NTR + CK	5.55 ± 1.27e	17.88 ± 4.34d	92.21 ± 1.52c	24.88 ± 0.4e	13.77 ± 0.79f	21.52 ± 1.62d
230 d	DPR + M44	28.43 ± 0.05a	47.24 ± 0.69a	95 ± 1.79a	38.36 ± 1.82a	31.73 ± 0.67a	22.57 ± 0.86ab
	DPR + CK	14.9 ± 0.73d	23.6 ± 0.19c	73.21 ± 1.37d	37.65 ± 3.12ab	26.62 ± 0.31c	17.55 ± 0.14d
	SSR + M44	22.57 ± 0.22b	46.33 ± 0.99a	92.91 ± 3.04a	36.36 ± 0.67ab	29.26 ± 0.51b	22.85 ± 0.11a
	SSR + CK	13.31 ± 1.87d	21.31 ± 2.58 cd	78.8 ± 0.49c	36.15 ± 0.74abc	23.94 ± 0.29d	18.99 ± 0.47c
	NTR + M44	20.25 ± 1.16c	39.85 ± 1.56b	83.85 ± 1.25b	33.27 ± 0.53c	21.19 ± 1.09e	21.7 ± 0.65b
	NTR + CK	7.71 ± 1.01e	19.95 ± 1.04d	74.25 ± 1.57d	34.73 ± 0.71bc	16.07 ± 0.51f	16.84 ± 0.88d
275 d	DPR + M44	39.01 ± 1.59a	49.5 ± 0.13a	99.49 ± 0.98a	26.78 ± 0.64a	36.1 ± 0.72a	24.17 ± 0.7bc
	DPR + CK	16.11 ± 0.15d	32.4 ± 0.54c	81.1 ± 1.38c	25.91 ± 0.4a	32.59 ± 0.46c	22.57 ± 0.38de
	SSR + M44	32.81 ± 0.16b	48.72 ± 3.72a	97.5 ± 1.88a	24.72 ± 0.92b	34.13 ± 0.41b	29.96 ± 1.18a
	SSR + CK	15.48 ± 0.29d	31.04 ± 1.04c	90.34 ± 2.77b	23.85 ± 0.28b	31.58 ± 0.45 cd	25.25 ± 0.43b
	NTR + M44	25.61 ± 0.61c	41.86 ± 0.54b	92.65 ± 1.64b	22.75 ± 0.76c	31.31 ± 0.37d	23.32 ± 0.3 cd
	NTR + CK	13.57 ± 0.1e	22.38 ± 3.99d	82.5 ± 1.36c	22.08 ± 0.41c	29.11 ± 0.88e	21.42 ± 1.05e
320 d	DPR + M44	28.47 ± 0.45a	31.67 ± 0.75a	87.43 ± 2.45a	23.73 ± 2.04ab	19.18 ± 0.78a	28.1 ± 0.67a
	DPR + CK	13.15 ± 0.15c	13.07 ± 0.74c	77.58 ± 1.05c	26.22 ± 2.26a	15.23 ± 0.77c	27.55 ± 0.6a
	SSR + M44	17.79 ± 0.95b	28.16 ± 1.37b	91.45 ± 1.47b	21.92 ± 2.79bc	16.98 ± 0.5b	28.39 ± 0.8a
	SSR + CK	10.12 ± 0.75d	11.44 ± 0.65d	79.15 ± 0.71c	24.04 ± 1.61ab	14.41 ± 0.31c	24.59 ± 1.79b
	NTR + M44	11.03 ± 0.56d	14.25 ± 0.47c	79.63 ± 1.22c	20.65 ± 0.45bc	14.64 ± 1c	20.25 ± 1.02c
	NTR + CK	8.78 ± 0.22e	10.82 ± 0.27d	76.88 ± 1.97c	19.99 ± 0.22c	12.14 ± 0.41d	15.9 ± 0.23d

Under identical conditions during the same period, different lowercase letters following the same column of numbers indicate significant differences between treatments (*P* < 0.05).

### 3.3 Effects of straw return methods with inoculant application on soil bacterial community

**Figure 4:**
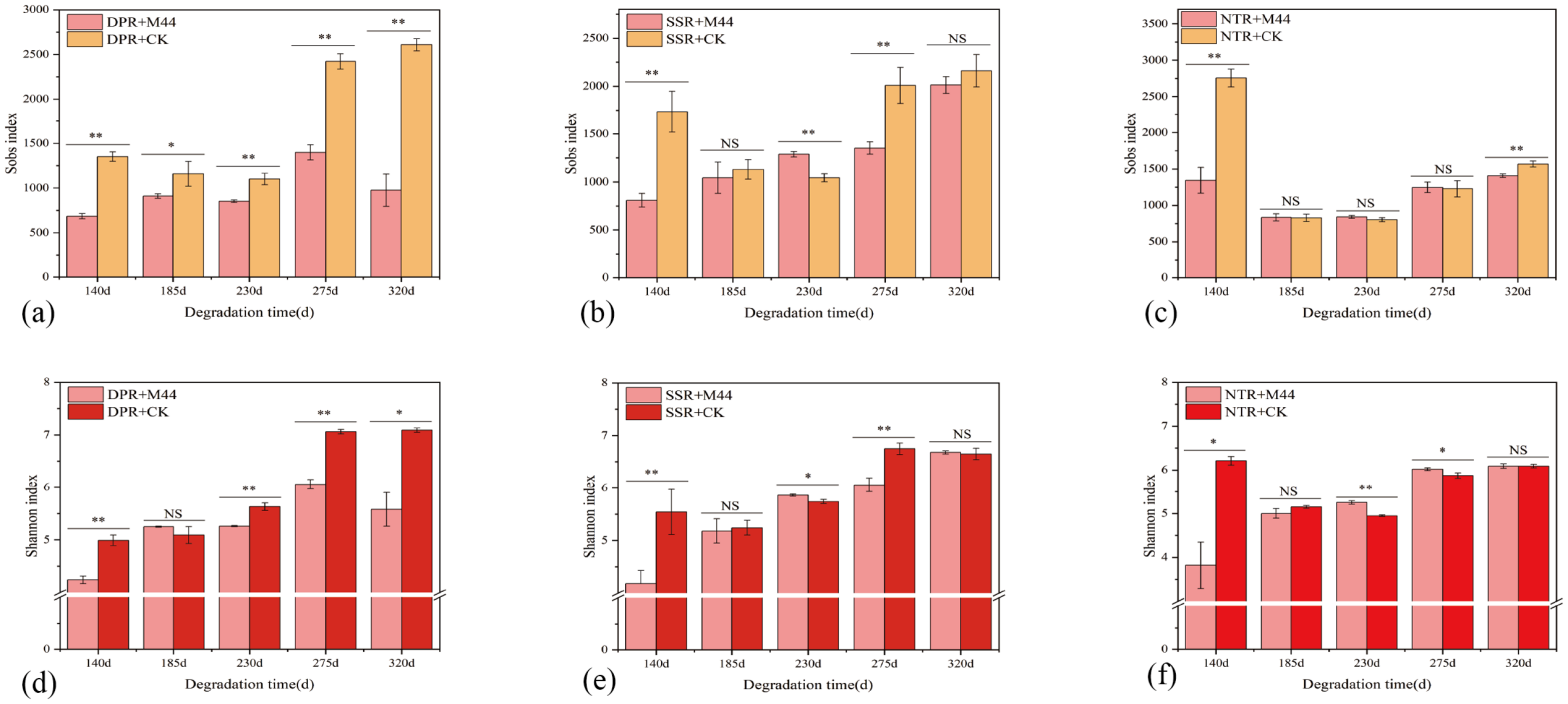
Sobs **(a–c)** and Shannon **(d–f)** indices under different straw return methods with inoculant application across sampling periods. * and ** indicate significance at *P* < 0.05 and *P* < 0.01 levels, respectively; NS indicates non-significant effects.

Compared with the non-inoculated treatment, DPR + M44 significantly decreased the Sobs index and Shannon index at all sampling stages (except 185 d), with reductions of 247.00–1635.00 and 0.37–1.51, respectively (Figures 4a,d). SSR + M44 significantly decreased the Sobs index and Shannon index at 140, 230, and 275 d, with reductions of 86.67–926.00 and 0.06–1.36, respectively (Figures 4b,e). NTR + M44 only significantly decreased the Sobs index and Shannon index at 140 d, with reductions of 1413.00 and 2.39, respectively (Figures 4c,f). Moreover, the temporal variation trends of the Sobs index and Shannon index in the DPR, SSR, and NTR treatments were generally consistent with those of soil extracellular enzyme activity. Therefore, it is suggested that the application of inoculant M44 may accelerate straw decomposition by enriching functional microbial communities related to straw degradation and further promoting the secretion of hydrolytic enzymes.

**Figure 5:**
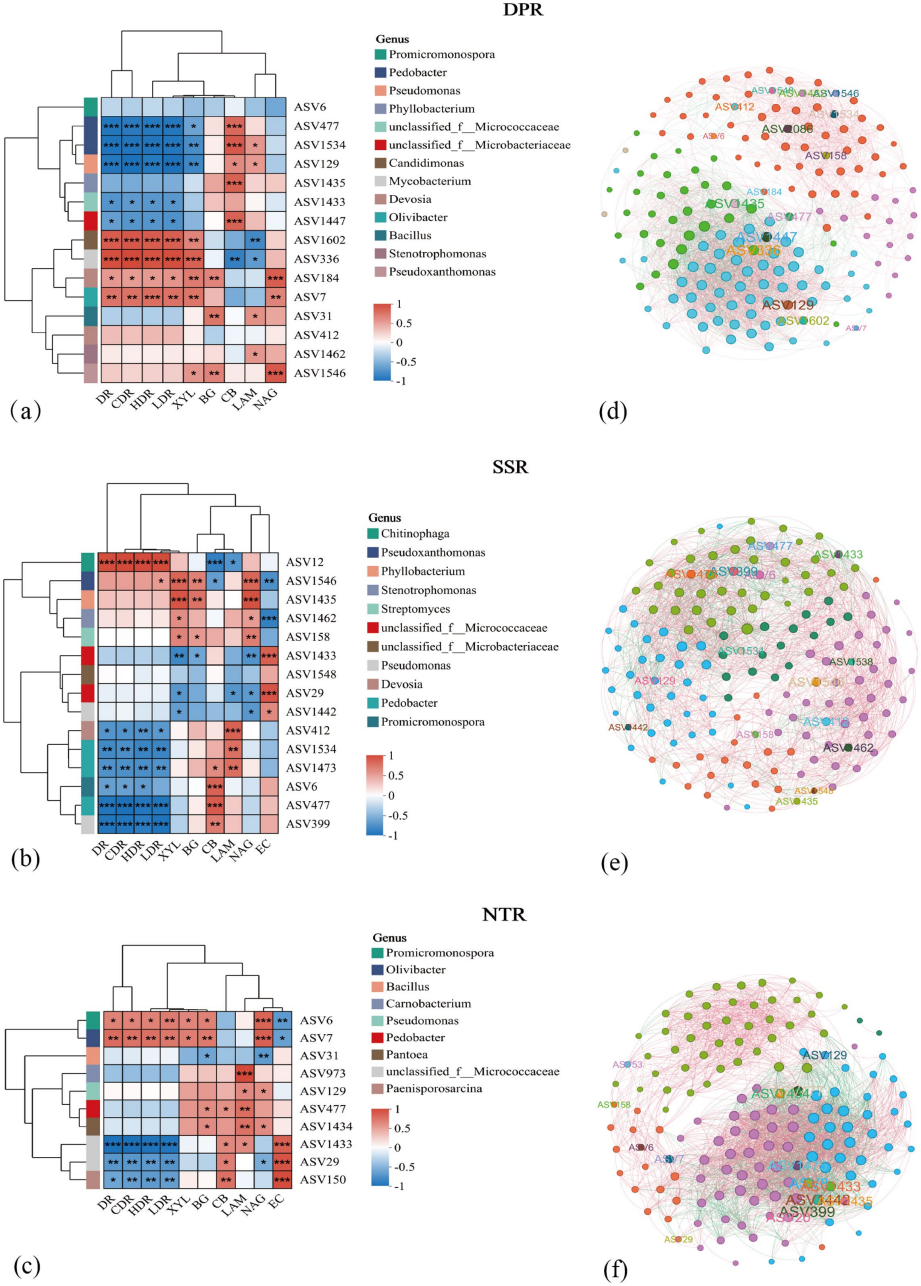
Screening of key amplicon sequence variants (ASVs) across treatments. **(a–c)** Correlation analysis between key ASVs and enzyme activities and straw degradation indices. DR, Straw degradation rate; CDR, cellulose degradation rate; HDR, hemicellulose degradation rate; LDR, lignin degradation rate; XYL, β-1,4-xylosidase; BG, β-1,4-glucosidase; CB, β-D-glucanase; LAM, Leucine aminopeptidase; NAG, acetylglucosaminidase; EC, Laccase. *, **, and *** indicate significance at *P* < 0.05, *P* < 0.01, and *P* < 0.001 levels, respectively. (d–f) Correlation networks of key ASVs.

To identify the core functional taxa, key sequences were first screened at the ASV level; a total of 63,038 operational taxonomic units (ASVs) from the main effect of each tillage treatment were standardized, and ASVs with relative abundance ≥0.1% in samples were selected, finally retaining 140, 137, and 154 ASVs for DPR, SSR, and NTR treatments, respectively (see Supplementary material: Significant ASVs _taxon_asv.full.xlsx); based on the integrated ASV dataset for each tillage treatment, species succession analysis was further performed, the top 30 ASVs by abundance were selected at the taxonomic level for visualization, and according to their enrichment during straw degradation, 15, 15, and 10 key ASVs were finally identified for DPR, SSR, and NTR treatments, respectively (Supplementary Figures S4–S6); a co-occurrence network was constructed, and network analysis showed that these key ASVs were located in the core positions of the network modules (Figures 5d–f); combined with the succession dynamics and taxonomic annotation, we found that under inoculant application, these key ASVs mainly belonged to several genera with known degradation functions (see Supplementary Table S1); finally, correlation analysis was performed between the screened key ASVs and parameters of straw degradation and hydrolytic enzyme activities (Figures 5a–c), and we ultimately identified the core functional taxa regulated by inoculant M44 at the ASV level under different straw returning patterns: in the DPR treatment, ASV129, belonging to Pseudomonas, was enriched in the early stage and showed a significantly positive correlation with β-cellobiosidase activity; ASV477 and ASV1534, belonging to Pedobacter, were enriched in the middle stage and significantly positively correlated with β-cellobiosidase activity; ASV184 and ASV412, belonging to *Devosia*, were enriched in the late stage and significantly positively correlated with straw degradation indicators and β-xylosidase activity; in the SSR treatment, ASV1462 and ASV158, belonging to *Stenotrophomonas* and *Streptomyces*, respectively, were enriched throughout the whole stage and significantly positively correlated with β-xylosidase, β-glucosidase, and leucine aminopeptidase activities; ASV1546, belonging to *Pseudoxanthomonas*, was enriched intermittently and significantly positively correlated with β-xylosidase, β-glucosidase, and leucine aminopeptidase activities; in the NTR treatment, ASV6 and ASV7, belonging to *Promicromonospora* and *Olivibacter*, respectively, were enriched in the middle–late stage and significantly positively correlated with straw degradation and β-xylosidase, β-glucosidase, and leucine aminopeptidase activities; ASV31 and ASV1434, belonging to *Bacillus* and *Pantoea*, were enriched in the early–middle and middle–late stages, respectively.

**Figure 6:**
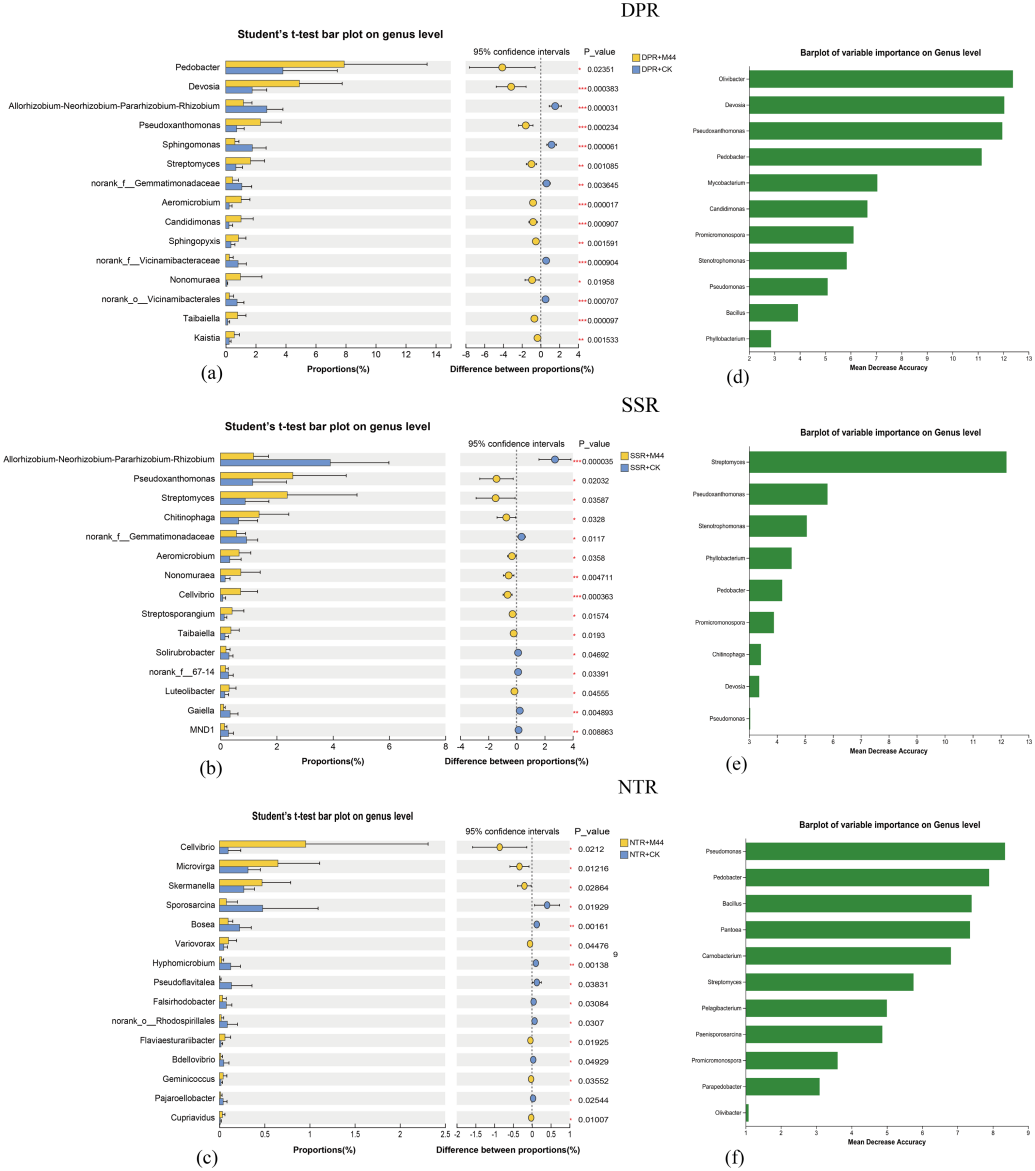
Screening of key bacterial genera across treatments. **(a–c)** Differential abundance of bacterial genera. **(d–f)** Random forest analysis of bacterial genera.

To verify the regulatory effect of inoculant M44 at a higher taxonomic level, differential species analysis (Student’s *t*-test) of the bacterial community was now performed at the genus level; based on the relative abundance ranking, the top 15 dominant genera with significant differences between groups were selected for visualization (*P* < 0.05); compared with the non-inoculated control, DPR + M44 significantly enriched genera such as *Pedobacter*, *Devosia*, *Pseudoxanthomonas*, *Streptomyces*, and *Taibaiella*; SSR + M44 significantly enriched genera such as *Pseudoxanthomonas*, *Streptomyces*, *Chitinophaga*, and *Nonomuraea*; NTR + M44 significantly increased the abundance of genera such as Cellvibrio and Microvirga (Figures 6a–c); a Random Forest model was used at the genus level to screen core predictive species; based on the model importance score, the top 15 key bacterial genera were selected as core predictors to identify marker bacterial genera highly related to the experimental treatments and further verify the reliability of the differential species analysis results; the results showed that genera such as *Olivibacter*, *Devosia*, *Pseudoxanthomonas*, *Pedobacter*, *Promicromonospora*, *Stenotrophomonas*, and *Pseudomonas* were strong predictors for the DPR + M44 treatment; genera such as *Streptomyces*, *Pseudoxanthomonas*, *Stenotrophomonas*, *Pedobacter*, *Promicromonospora*, *Chitinophaga*, and *Devosia* were strong predictors for the SSR + M44 treatment; and genera such as *Pseudomonas*, *Pedobacter*, *Bacillus*, *Pantoea*, *Carnobacterium*, *Streptomyces*, and *Promicromonospora* were strong predictors for the NTR + M44 treatment (Figures 6d–f); this also means that the application of inoculant M44 significantly regulated the abundance of the above genera under DPR, SSR, and NTR conditions.

Finally, ROC analysis was performed on the selected key ASVs and key genera to verify and evaluate the accuracy of the results; the results showed that the AUC values of the selected key ASVs and key genera in the DPR + M44, SSR + M44, and NTR + M44 treatments were 0.77, 0.71, 0.60 and 0.64, 0.73, 0.64, respectively, indicating that the screening results had high accuracy, and the prediction effect of the DPR and SSR treatments was better than that of the NTR treatment (see Supplementary Figures S7–S12); in summary, inoculant M44 drives the straw degradation process by differentially and directionally enriching specific functional genera such as *Pedobacter*, *Streptomyces*, *Pseudoxanthomonas*, *Olivibacter*, and *Devosia* (and their corresponding ASVs) under different straw returning patterns, thereby regulating the soil bacterial community structure.

### 3.4 Effects of straw return methods with inoculant application on functional predictions

**Figure 7:**
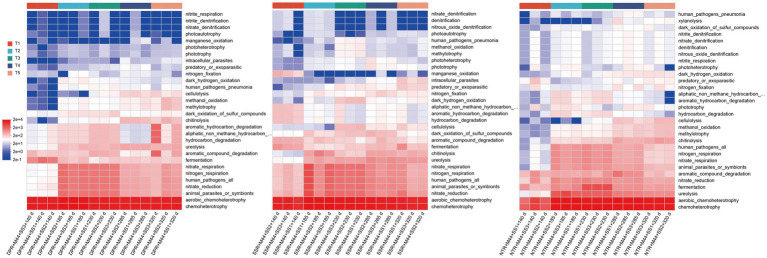
Effects of different straw return methods with inoculant application on bacterial community functions across degradation stages. T1, T2, T3, T4, and T5 represent 140, 185, 230, 275, and 320 days, respectively.

To further clarify the differential regulation of soil microbial metabolic functions by the application of microbial inoculant M44 under different straw returning practices, a predictive analysis of the metagenomic functional profile was conducted based on the FAPROTAX database; functional annotation results showed that chemoheterotrophy and aerobic chemoheterotrophy maintained high relative abundances throughout the entire degradation cycle across all treatments (Figure 7), indicating that chemoheterotrophic metabolism is the core pathway for soil microorganisms to acquire energy, providing fundamental driving force for straw degradation; regarding nitrogen transformation functions, nitrate respiration and nitrate reduction also exhibited consistently high abundance throughout the entire period, indicating that all three straw returning methods can drive the reductive transformation of soil nitrogen and maintain the basic framework for nitrogen form turnover; in addition, the abundances of fermentation and chitinolysis functions were prominent, which may be related to the local microenvironment formed around the straw promoting the activity of fermentative microorganisms and the chitin substrates brought by the straw inducing the proliferation of related microorganisms; in terms of the degradation functions of major straw components, the three returning patterns showed significant differentiation (Figure 7); in the DPR + M44 treatment, the abundances of cellulolysis, aromatic_hydrocarbon_degradation, aliphatic_non_methane_hydrocarbon_degradation, nitrogen_respiration, and ureolysis were significantly higher than those in other treatments (*P* < 0.05), indicating that this treatment has advantages in the degradation of specific aromatic hydrocarbons, the breakdown of aliphatic carbon chains, and urea decomposition, thereby accelerating the conversion of nitrogen from organic to inorganic forms; the SSR + M44 treatment exhibited a significantly higher abundance of aromatic_compound_degradation (*P* < 0.05), indicating that it focuses more on the overall degradation of complex aromatic carbon structures such as lignin; the NTR + M44 treatment had significantly higher functional abundances of methanol_oxidation and methylotrophy compared to the DPR + M44 and SSR + M44 treatments (*P* < 0.05), suggesting that specific substances released from the straw under this treatment directionally enriched methylotrophic microorganisms, enhancing the ability of diversified carbon source utilization; in summary, the three straw returning methods combined with microbial inoculant M44 showed commonalities in basic metabolic functions (e.g., chemoheterotrophy, nitrate reduction) but significant differences in key functions such as straw degradation, carbon metabolism, and nitrogen transformation; these differences stem from changes in soil microenvironment and substrate composition induced by straw returning methods, providing important functional-level evidence for optimizing straw returning practices.

### 3.5 Microbial decay-promoting mechanisms under different straw return methods with inoculant application

**Figure 8:**
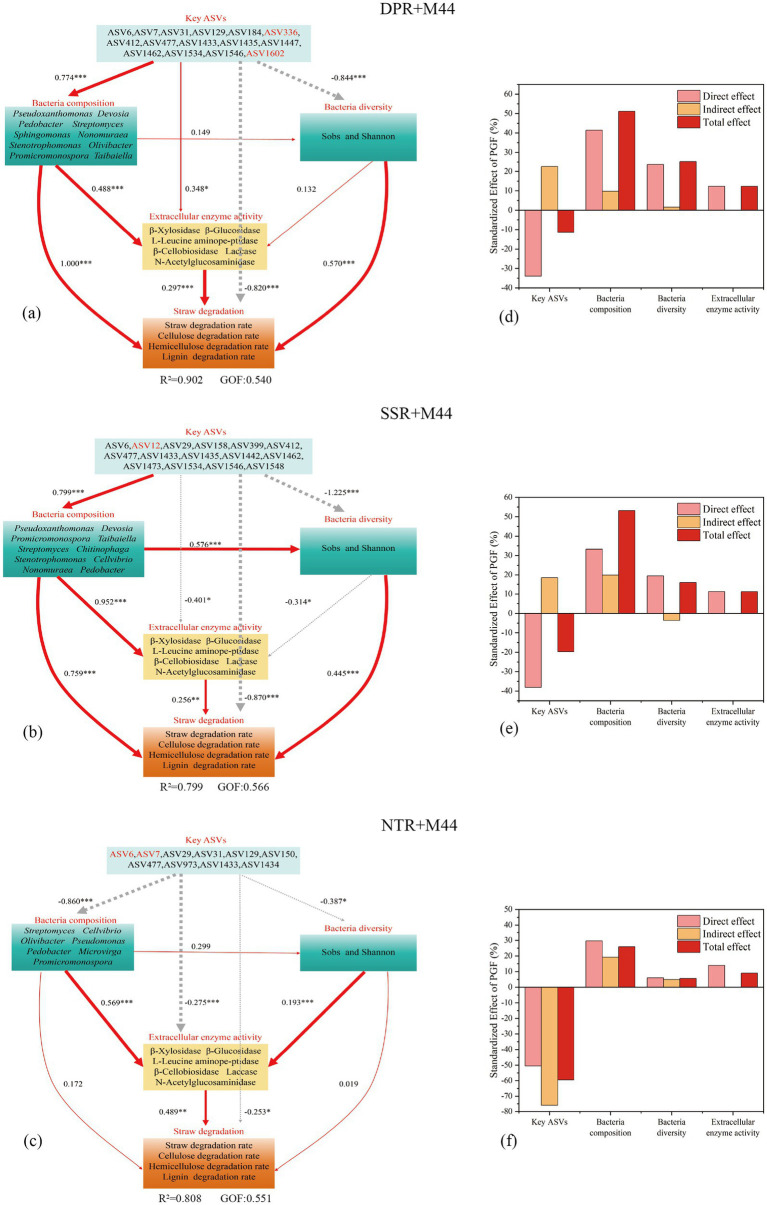
Partial least squares path modeling results and effect coefficients: elucidating the mechanisms by which different straw return methods with inoculant application influence straw degradation through bacterial key ASVs, composition, diversity, and extracellular enzyme activities. Red and gray lines represent positive and negative correlations, respectively. Arrow width indicates relationship strength, with wider arrows denoting stronger effects. Numbers adjacent to lines are standardized path coefficients. *, **, and *** indicate significance at *P* < 0.05, *P* < 0.01, and *P* < 0.001 levels, respectively. **a–c** are partial least squares path modeling (PLS-PM) diagrams; **d–f** are bar charts of the standardized effect of PGF (%).

To reveal the microbial driving mechanisms by which the microbial inoculant M44 promotes straw decomposition under different straw returning methods, this study constructed partial least squares path models (PLS-PM). All treatment models exhibited good model fit (GOF = 0.540–0.566) (Figure 8).

Path analysis results showed that in the DPR + M44 treatment, bacterial composition (*Pseudoxanthomonas*, *Devosia*, *Streptomyces*, etc.) was the most important driving factor for straw degradation (*r* = 1.000, *P* < 0.001), contributing 51.12% to the total degradation effect; followed by bacterial diversity (Sobs and Shannon indices) (contributing 25.21% to the total degradation effect, *r* = 0.570, *P* < 0.001) and extracellular enzyme activity (β-xylosidase, β-glucosidase, etc.) (contributing 12.29% to the total degradation effect, *r* = 0.297, *P* < 0.001). In contrast, the key bacterial ASVs (ASV6, ASV7, ASV31, etc.) indirectly participated in straw degradation by directly regulating bacterial composition (*r* = 0.774, *P* < 0.001) and extracellular enzyme activity (*r* = 0.348, *P* < 0.05) (Figures 8a,d).

The SSR + M44 treatment exhibited similar microbial driving mechanisms to the DPR + M44 treatment, where bacterial composition (*Pseudoxanthomonas*, *Streptomyces*, *Chitinophaga*, etc.) remained the core driving factor for straw degradation (contributing 53.13% to the total effect, *r* = 0.759, *P* < 0.001), followed by bacterial diversity (*r* = 0.445, *P* < 0.001) and extracellular enzyme activity (*r* = 0.256, *P* < 0.01), which contributed 15.94 and 11.21% to the total effect on straw degradation, respectively; the key bacterial ASVs (ASV6, ASV12, ASV29) directly and positively drove bacterial composition (*r* = 0.799, *P* < 0.001), which in turn indirectly regulated bacterial diversity (*r* = 0.576, *P* < 0.001) and extracellular enzyme activity (*r* = 0.952, *P* < 0.001), thereby indirectly participating in straw degradation (Figures 8b,e).

In contrast, under the NTR + M44 treatment, extracellular enzyme activity emerged as the most important driver of straw degradation (*r* = 0.489, *P* < 0.001, total effect contribution 9.00%). This activity was directly regulated by bacterial composition (*Streptomyces*, *Cellvibrio*, *Olivibacter*, etc.) (*r* = 0.569, *P* < 0.001) and bacterial diversity (*r* = 0.193, *P* < 0.001). The total effect contributions of bacterial composition and diversity to straw degradation were 25.90 and 5.60%, respectively (Figures 8c,f).

In summary, the PLS-PM model revealed distinct pathways of microbial-driven straw degradation by inoculant M44 under different straw returning practices. The DPR and SSR treatments formed a hierarchical driving chain of “key ASV enrichment-functional microbial community enrichment -increased extracellular enzyme activity-enhanced straw degradation” during straw degradation, whereas the NTR treatment exhibited a straw degradation pattern primarily driven by “increased extracellular enzyme activity” and indirectly influenced by “bacterial composition and bacterial diversity.” This finding clarifies the hierarchical relationship where “key ASVs serve as higher-level regulatory units, key bacterial genera act as direct functional executors, extracellular enzyme activity functions as the operational tool, and straw degradation represents the ultimate outcome.” It also provides a microbial-driven theoretical basis for the precise optimization of straw decomposition promotion strategies using microbial inoculant M44 under different straw returning practices.

## 4 Discussion

By integrating field tillage practices with an improved *in situ* nylon bag microcosm degradation test, this study systematically elucidated the microbial mechanisms by which the composite microbial inoculant M44 drives corn straw degradation under three straw returning practices—deep plowing and returning to soil (DPR), deep loosening and mixing with soil (SSR), and no-till mulching (NTR). The results showed that the combined application of M44 inoculant under different tillage practices differentially and directionally enriched specific functional bacterial genera (e.g., *Pseudoxanthomonas*, *Devosia*, *Streptomyces*, *Olivibacter*, *Pedobacter*) and their key ASVs (e.g., ASV6, ASV12, ASV412, ASV1546), thereby reshaping the soil bacterial community structure, further significantly enhancing soil extracellular enzyme activities (e.g., β-xylosidase, β-glucosidase, β-cellobiosidase), and ultimately accelerating straw decomposition.

The decomposition rate of straw is affected by multiple factors, with local regional factors being more critical in small-scale areas. Tillage practices regulate the degree of contact between straw and soil, microbial colonization, and co-metabolic processes by affecting the returning depth (Hewins et al., 2013; Shahbaz et al., 2017). Yang F. K. et al. (2020), Yang X. R. et al. (2020) and Zhao et al. (2024) confirmed that straw decomposition inoculants can effectively promote the degradation of cellulose, hemicellulose, and lignin, thereby accelerating straw decomposition. This study confirmed that the M44 inoculant significantly increased the straw degradation rate by 2.33–9.81 percentage points (*P* < 0.05) in the temperate continental semi-arid climate zone (Figure 1a) and shortened the half-life (*t*_0.5_) by 20.7–62.8 days (Figure 1d), with a particularly significant improvement effect during the low-temperature period (0–185 days). This overcomes the limitation of microbial inoculant inactivation due to low temperature and drought in temperate continental climate zones reported by Yang F. K. et al. (2020), Yang X. R. et al. (2020) and Tao et al. (2024). There were significant differences in the decomposition-promoting effects among different straw returning methods: the straw degradation efficiency of the DPR and SSR treatments was significantly better than that of the NTR treatment (Figures 1a, 2a), which is consistent with the “tillage-dominated” conclusion of Wang et al. (2021), but the tillage advantage was further amplified through the synergy of the inoculant. This variation mainly stems from the regulation of soil microenvironment and microbial-straw interaction interface by different tillage practices combined with inoculant application (Wei et al., 2024; Pei et al., 2023): DPR and SSR increased oxygen and water permeability by deeply disturbing the soil, promoted full contact between straw and indigenous microorganisms as well as inoculated microbial agents, and created more favorable conditions for the colonization of functional microbial communities and enzymatic reactions (Qiu et al., 2025). In contrast, NTR no-till practice resulted in straw retention on the soil surface, leading to significant fluctuations in thermal and moisture conditions, exacerbating low-temperature and drought stresses, limiting microbial colonization efficiency, and thereby weakening the direct effect of the inoculant (Zhang M. et al., 2025). The dense microbial colonies and pores on the surface of straw in the inoculated treatment in the scanning electron microscopy (SEM) images (Figure 2b) more intuitively proved that this optimized physical interface promoted microbial adhesion and colonization (Sarma et al., 2022).

The microbial inoculant M44 significantly enhanced the activity of hydrolytic enzymes such as β-xylosidase and β-glucosidase (Table 2), with the comprehensive enzyme index increasing by 0.74–1.06 (*P* < 0.05) (Figure 3). Compared with the conclusion on enzyme activity promotion drawn by Song et al. (2023), this study further found that the enzyme activity response of the DPR and SSR treatments was better than that of the NTR treatment. This is primarily attributed to changes in soil physical and nutrient conditions under different straw returning practices, which directly or indirectly drive the succession of soil microbial community structure, thereby enhancing the activity expression of key enzymes involved in straw degradation (Wang et al., 2016; Guo et al., 2018). Notably, the application of the M44 inoculant reduced bacterial α-diversity (Sobs and Shannon indices) in this study (Figure 4), and the M44 inoculant exerted its effect by enriching key functional bacteria (e.g., *Pedobacter* and *Devosia* in DPR; *Pseudoxanthomonas* and *Streptomyces* in SSR; *Cellvibrio* in NTR) and restructuring the community structure (Figure 6). This enrichment primarily resulted from the inoculant-induced alteration of soil microbial community structure and the diverse nutrients supplied by the returned straw, which promoted the proliferation of straw-degrading functional microbial communities (Huang et al., 2025). This aligns with the findings by Kimeklis et al. (2025) that changes in soil extracellular enzyme activity can reflect the succession patterns of soil microbial communities during straw degradation. Network analysis in this study revealed that these key ASVs occupy central positions in microbial interaction networks (e.g., ASV129 (*Pseudomonas*), ASV477 (*Pedobacter*), ASV184 (*Devosia*) in DPR; ASV1462 (*Stenotrophomonas*), ASV158 (*Streptomyces*) in SSR; ASV6 (*Promicromonospora*) and ASV7 (*Olivibacter*) in NTR), and showed significant positive correlations with key hydrolytic enzyme activities (Figure 5). This association indicates that specific functional microorganisms directly drive the decomposition of straw components by secreting or activating degradative enzymes (Liu D. et al., 2025; Liu Q. M. et al., 2025; Chen et al., 2025). Previous studies from our laboratory have confirmed that the core strains of M44 (e.g., *Devosia*, *Stenotrophomonas*, *Pseudomonas*) possess corresponding enzymatic functional potentials (e.g., cellulase, xylanase, laccase) (Zhang et al., 2022), thereby exhibiting the capacity to degrade lignocellulose and xylan in straw (Wang L. J. et al., 2025; Wang Y. Q. et al., 2025; Sunardi and Istikowati, 2020). This study further clarifies the core role of the microbial inoculant M44 in community succession and its coupling relationship with enzyme activity under field conditions. This demonstrates that the application of exogenous microbial inoculants promotes synergistic interactions with indigenous microorganisms, where the successful colonization and restructuring of the microbial community facilitate the production of hydrolytic enzymes and straw degradation (Zhang Y. F. et al., 2025; Junior et al., 2020; Mula-Michel and Williams, 2013).

Functional prediction analysis indicates (Figure 7) that DPR + M44 exhibits superior performance in cellulolysis and aromatic hydrocarbon degradation; SSR + M44 excels in the degradation of complex aromatic compounds; while NTR + M44 enriches methylotrophic microorganisms. These functional differences reflect the distinct shaping of soil substrate composition and microenvironments by different straw returning methods combined with M44 inoculant application (Fu et al., 2025; Jensen et al., 2021).

Notably, the pathways through which the microbial inoculant M44 drives straw degradation differ across various straw returning methods. Partial Least Squares Path Model (PLS-PM) analysis showed that in the DPR and SSR treatments, bacterial community composition (e.g., *enriched Pseudoxanthomonas*, *Devosia*, *Streptomyces*) was the most important direct driver of degradation, followed by bacterial diversity (Sobs, Shannon index) and extracellular enzyme activity (β-glucosidase, β-cellobiosidase, β-xylosidase, etc.). Key ASVs (e.g., ASV6, ASV12, ASV412) indirectly influenced the degradation process by regulating bacterial composition and enzyme activity. In the NTR treatment, minimal tillage disturbance limited microbial community reorganization, making extracellular enzyme activity the core driver of degradation, and this activity was directly driven by bacterial composition and diversity Zhang Z. D. et al. (2025). This finding clarifies the differential expression of the hierarchical interaction mechanism “key microorganisms- bacterial community structure- extracellular enzyme activity-straw degradation” when combined with M44 inoculant application under different tillage practices, providing a new perspective for understanding the mechanism of action of the M44 inoculant in complex field environments. Compared to previous studies predominantly conducted under controlled laboratory conditions or single straw returning methods (Cheng et al., 2021; Piao et al., 2023; Sui et al., 2021), this study is the first to elucidate the microbial-enzyme activity interaction regulation mechanism under the multifactorial (tillage-inoculant) interaction in the field: the DPR and SSR treatments enhanced oxygen and water permeability, prompting *Pseudoxanthomonas*, *Devosia*, and others to activate enzyme systems through quorum sensing (Zhang et al., 2024), thereby achieving straw degradation (contribution rate: 51.12%). In contrast, the NTR treatment restricted microbial community activity due to low-temperature drought caused by no-till practices (Wang L. J. et al., 2025; Wang Y. Q. et al., 2025; Phukongchai et al., 2022; Liu D. et al., 2025; Liu Q. M. et al., 2025), instead relying on the direct action of extracellular enzyme activity to degrade straw (contribution rate: 48.9%).

In summary, this study established the first “tillage-biocontrol agent” interaction model in spring maize-growing sandy loam soils of Inner Mongolia. By regulating the soil microenvironment to directionally shape functional microbial communities and activate extracellular enzyme systems, it revealed a microbial-level interaction mechanism (key microorganisms-bacterial community structure -extracellular enzyme activity-straw degradation) to drive straw decomposition. This provides crucial theoretical and technical support for further optimizing “tillage-microbial inoculant” synergistic straw return technologies in temperate continental semi-arid regions.

## 5 Conclusion

In cold-arid regions, the application of the low-temperature degrading microbial inoculant M44 combined with straw return promotes the effective decomposition of returned straw through the hierarchical interaction mechanism of “key microorganisms-bacterial community structure-extracellular enzyme activity-straw degradation.” Under tillage-inoculant interaction conditions, regulating the soil microenvironment enables the targeted shaping and enrichment of key microbial groups with degradation functions (e.g., *Pseudoxanthomonas*, *Devosia*, *Streptomyces*, *Pseudomonas*, etc.) and their critical ASVs (e.g., ASV6, ASV12, ASV412, ASV1546, etc.), reshaping the soil bacterial community structure and significantly activating the soil extracellular enzyme activity system (e.g., β-glucosidase, β-xylosidase, etc., with the comprehensive enzyme index increasing by 0.74–1.06), thereby synergistically driving the rapid decomposition of returned straw. Compared to the untreated control, the application of the microbial inoculant M44 significantly increased the straw degradation rate by 2.33–9.81 percentage points and shortened the half-life by 20.7–62.8 days. However, the pathways through which M44 drives straw degradation differed across straw returning methods. In the DPR and SSR treatments, bacterial community composition was the most important direct driver of degradation, followed by bacterial diversity and extracellular enzyme activity, while key ASVs indirectly influenced the degradation process by regulating bacterial composition and enzyme activity. In contrast, extracellular enzyme activity became the core driver of degradation in the NTR treatment, and its activity was directly influenced by bacterial composition and diversity. These findings provide crucial scientific evidence for optimizing “tillage-microbial inoculant” synergistic straw resource utilization technologies in arid, semi-arid, and cold regions.

## Data Availability

The 16S rRNA gene amplicon sequencing raw data, biological sample information, and bioproject data presented in this study are deposited in the National Microbiology Data Center (NMDC) under the following accession numbers: bioproject: NMDC10020672, Biological samples: NMDC20570841, Raw sequencing data: NMDC40111408.
