# Supplementary material for: The application of straw returning combined with low-temperature degrading microbial inoculant M44 in cold and arid regions promotes the efficient decomposition of returned straw through the hierarchical interaction mechanism of “key microorganisms—bacterial community structure—extracellular enzyme activity—straw degradation”
Source: Front Microbiol. 2026 Apr 29;17:1765717. doi: 10.3389/fmicb.2026.1765717 (PMC13168190; doi:10.3389/fmicb.2026.1765717)
Supplement: Supplementary file 2 [file Table_1.docx]

supplementary material

The application of straw returning combined with low-temperature degrading microbial inoculant M44 in cold and arid regions promotes the efficient decomposition of returned straw through the hierarchical interaction mechanism of "key microorganisms - bacterial community structure - extracellular enzyme activity - straw degradation"


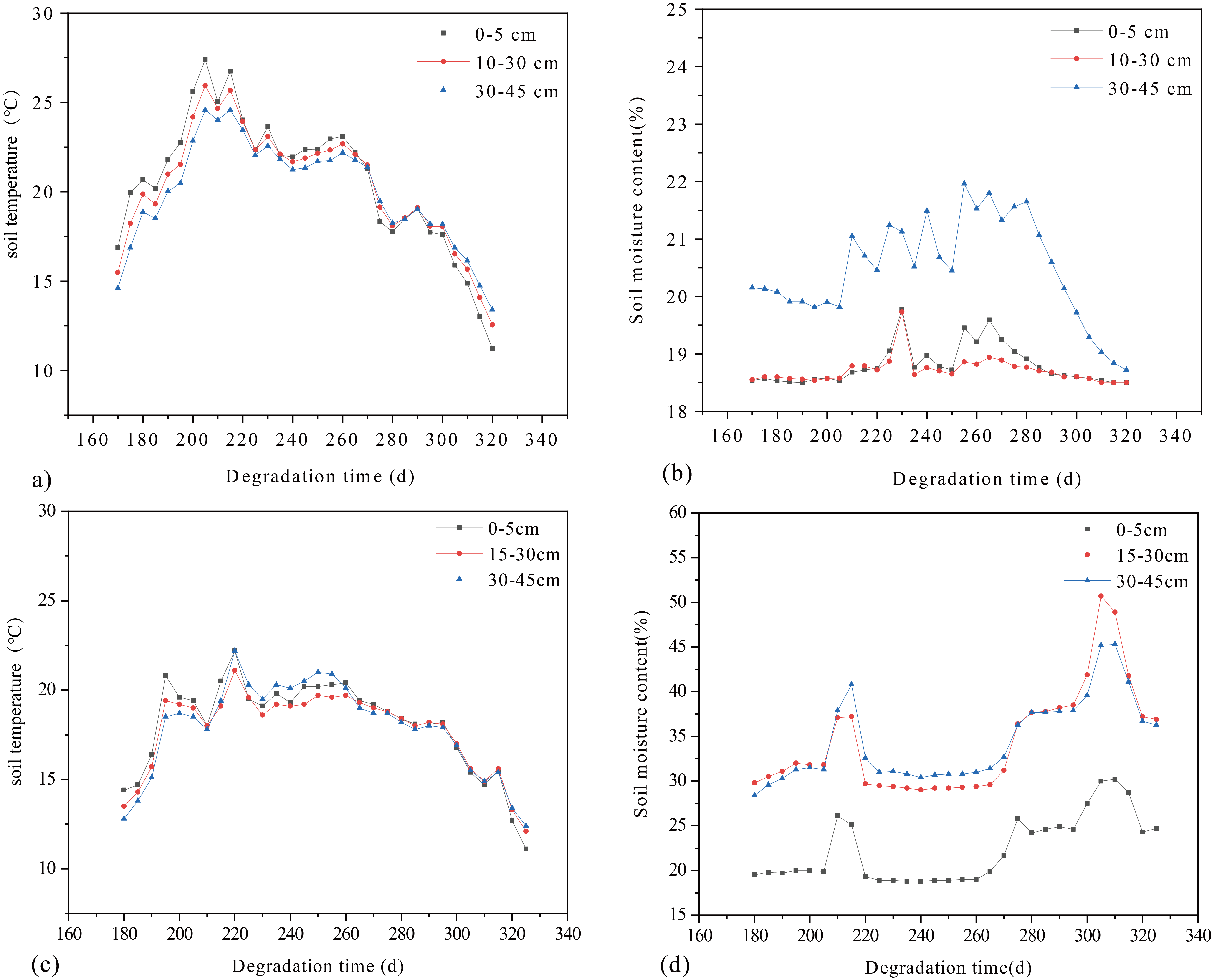


Fig. S1. Temporal variations in daily mean soil temperature and moisture (a-d) during straw degradation in 2023 and 2024. The soil depths monitored were 0–5, 10–30, and 30–45 cm, corresponding to the simulated tillage treatments in the field: no-tillage with straw mulching, deep subsoiling with mixed straw return, and deep ploughing with straw return, respectively.
