# Supplementary material for: The application of straw returning combined with low-temperature degrading microbial inoculant M44 in cold and arid regions promotes the efficient decomposition of returned straw through the hierarchical interaction mechanism of “key microorganisms—bacterial community structure—extracellular enzyme activity—straw degradation”
Source: Front Microbiol. 2026 Apr 29;17:1765717. doi: 10.3389/fmicb.2026.1765717 (PMC13168190; doi:10.3389/fmicb.2026.1765717)
Supplement: Supplementary file 3 [file Table_2.DOCX]

supplementary material

The application of straw returning combined with low-temperature degrading microbial inoculant M44 in cold and arid regions promotes the efficient decomposition of returned straw through the hierarchical interaction mechanism of "key microorganisms - bacterial community structure - extracellular enzyme activity - straw degradation"


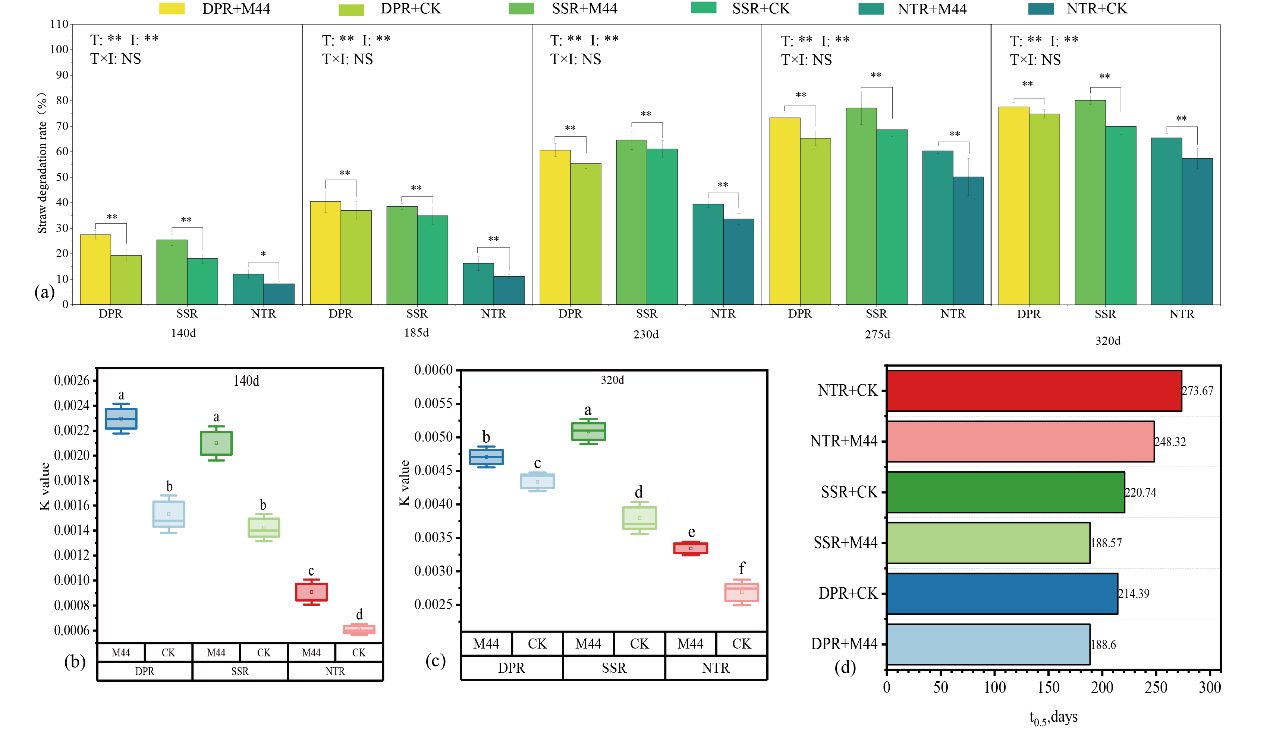


Fig. S2 Straw degradation efficiency under different straw return methods with microbial agent application. (a) Straw degradation rates across sampling periods under different straw return methods with microbial agent application. T and I represent tillage method and inoculation treatment, respectively; * and ** indicate significant effects at the 0.05 and 0.01 probability levels, respectively; NS indicates non-significant effects. (b-c) Decomposition constants at 140 d and 320 d under different straw return methods with microbial agent application. Different lowercase letters indicate significant differences among treatments (*P* < 0.05). (d) Half-life (t₀.₅, days) of straw degradation under different straw return methods with microbial agent application.

Table.S1. Straw degradation rates at different decomposition stages across treatments (%). Different lowercase letters within the same column indicate significant differences among treatments (*P* < 0.05).

| Treatment | Degradation time(d) | | | | | Fitted equation | R^2^ value |
| --- | --- | --- | --- | --- | --- | --- | --- |
|  | 0-140d | 140-185d | 185-230d | 230-275d | 275-320d |  |  |
| DPR-M44 | 27.47a | 12.98b | 20.23bc | 12.70bc | 4.40d | y = -0.82+191.42e^-0.0070x^ | 0.986 |
| DPR-CK | 19.27b | 17.63a | 18.53c | 9.85c | 9.71a | y = -0.89+197.45e^-0.0063x^ | 0.995 |
| SSR-M44 | 25.43a | 13.10b | 25.97a | 12.63bc | 3.17cd | y = 7.09+252.57e^-0.0094x^ | 0.999 |
| SSR-CK | 18.06b | 16.91ab | 26.03a | 7.69c | 1.48bcd | y =14.58+258.00e^-0.0095x^ | 0.966 |
| NTR-M44 | 11.92c | 4.26c | 23.25ab | 20.97a | 5.24bc | y=-92.58+246.23e^-0.0022x^ | 0.934 |
| NTR-CK | 8.16d | 2.93c | 22.53ab | 16.52ab | 7.39ab | y=-422.76+557.15e^-0.0006x^ | 0.998 |
