# Supplementary material for: The application of straw returning combined with low-temperature degrading microbial inoculant M44 in cold and arid regions promotes the efficient decomposition of returned straw through the hierarchical interaction mechanism of “key microorganisms—bacterial community structure—extracellular enzyme activity—straw degradation”
Source: Front Microbiol. 2026 Apr 29;17:1765717. doi: 10.3389/fmicb.2026.1765717 (PMC13168190; doi:10.3389/fmicb.2026.1765717)
Supplement: Supplementary file 4 [file Table_3.DOCX]

supplementary material

The application of straw returning combined with low-temperature degrading microbial inoculant M44 in cold and arid regions promotes the efficient decomposition of returned straw through the hierarchical interaction mechanism of "key microorganisms - bacterial community structure - extracellular enzyme activity - straw degradation"


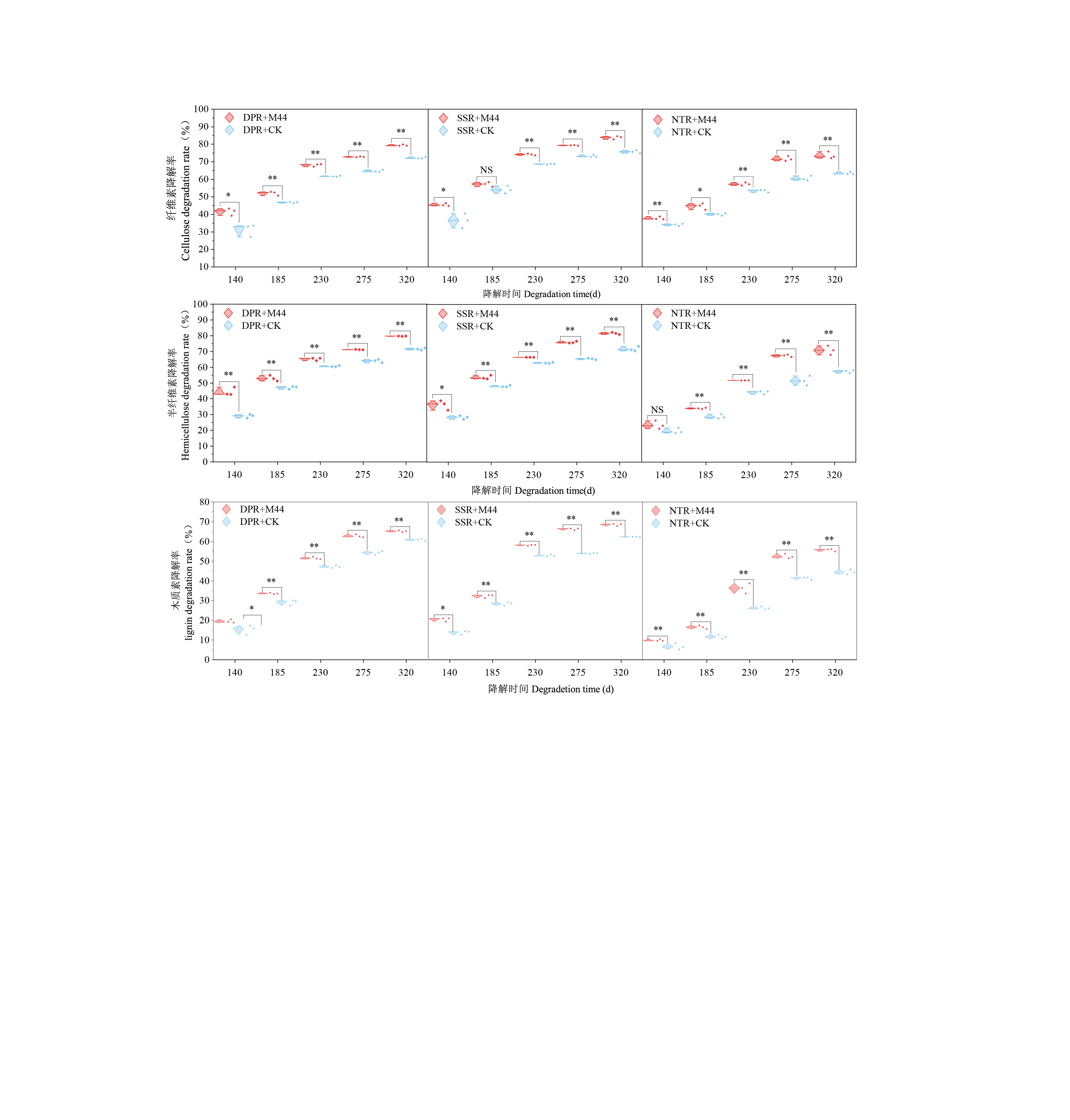


Fig. S3 Lignocellulose degradation rate of straw under different straw return methods with microbial agent application. * and ** indicate significance at *P* < 0.05 and *P* < 0.01 levels, respectively; NS indicates non-significant effects.
