# Supplementary material for: The application of straw returning combined with low-temperature degrading microbial inoculant M44 in cold and arid regions promotes the efficient decomposition of returned straw through the hierarchical interaction mechanism of “key microorganisms—bacterial community structure—extracellular enzyme activity—straw degradation”
Source: Front Microbiol. 2026 Apr 29;17:1765717. doi: 10.3389/fmicb.2026.1765717 (PMC13168190; doi:10.3389/fmicb.2026.1765717)
Supplement: Supplementary file 5 [file Table_4.docx]

supplementary material

The application of straw returning combined with low-temperature degrading microbial inoculant M44 in cold and arid regions promotes the efficient decomposition of returned straw through the hierarchical interaction mechanism of "key microorganisms - bacterial community structure - extracellular enzyme activity - straw degradation"


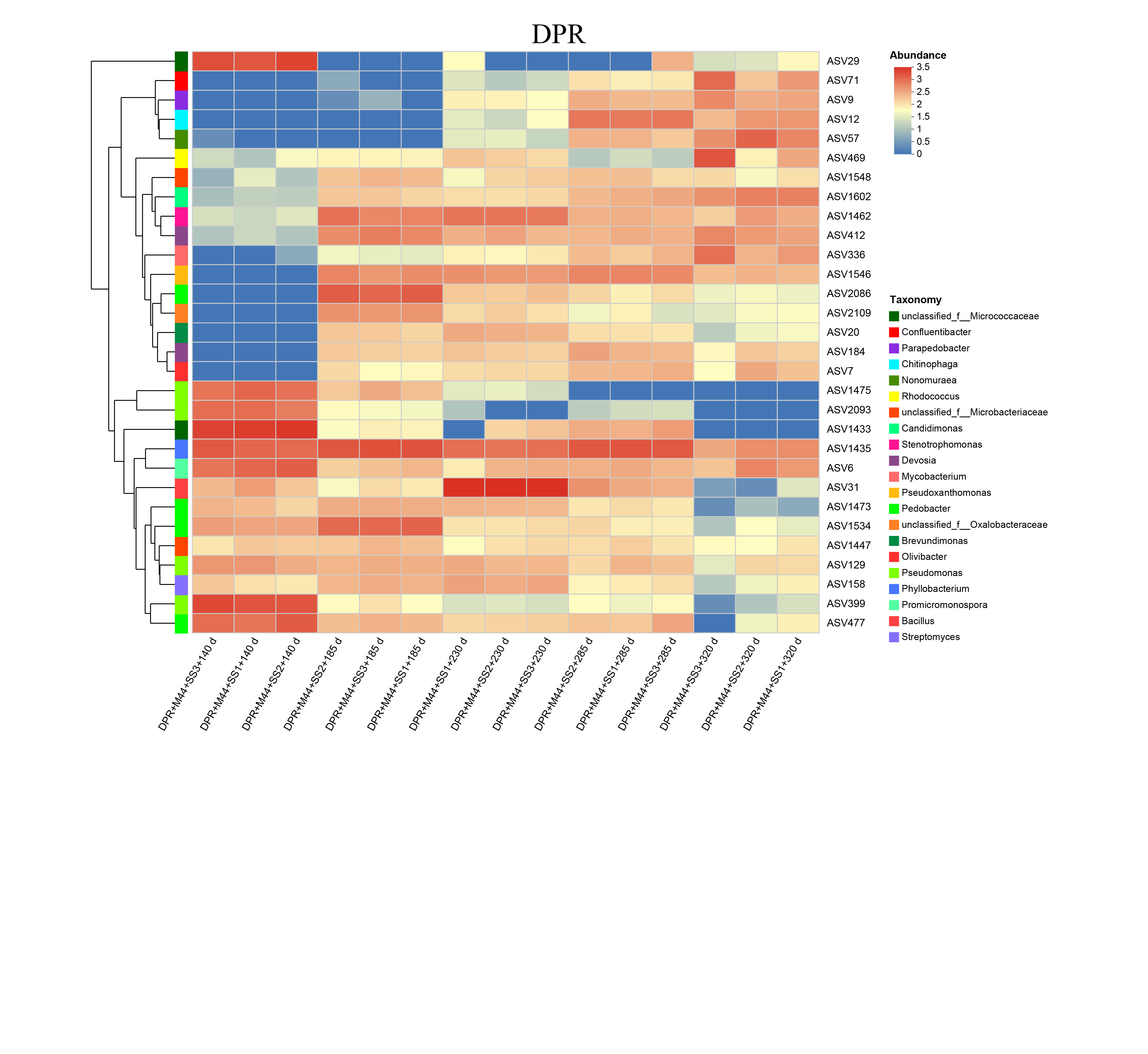


Fig S4. Temporal species succession of soil bacteria at the genus level during straw degradation in the deep tillage straw return treatment with microbial inoculant M44.


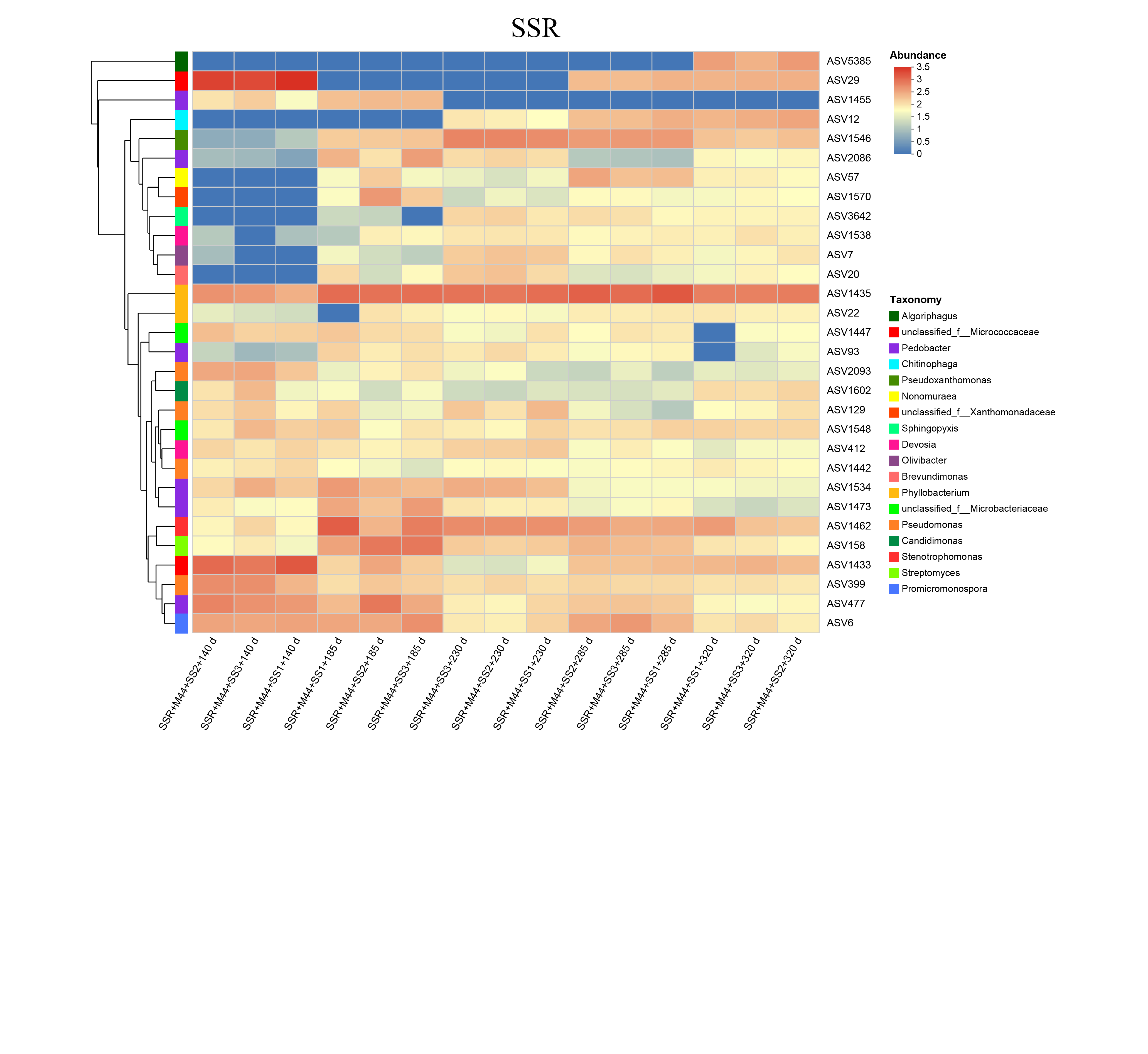


Fig S5. Analysis of species succession of soil bacteria at the genus level over degradation time under deep loosening mixed straw return with application of microbial inoculant M44.


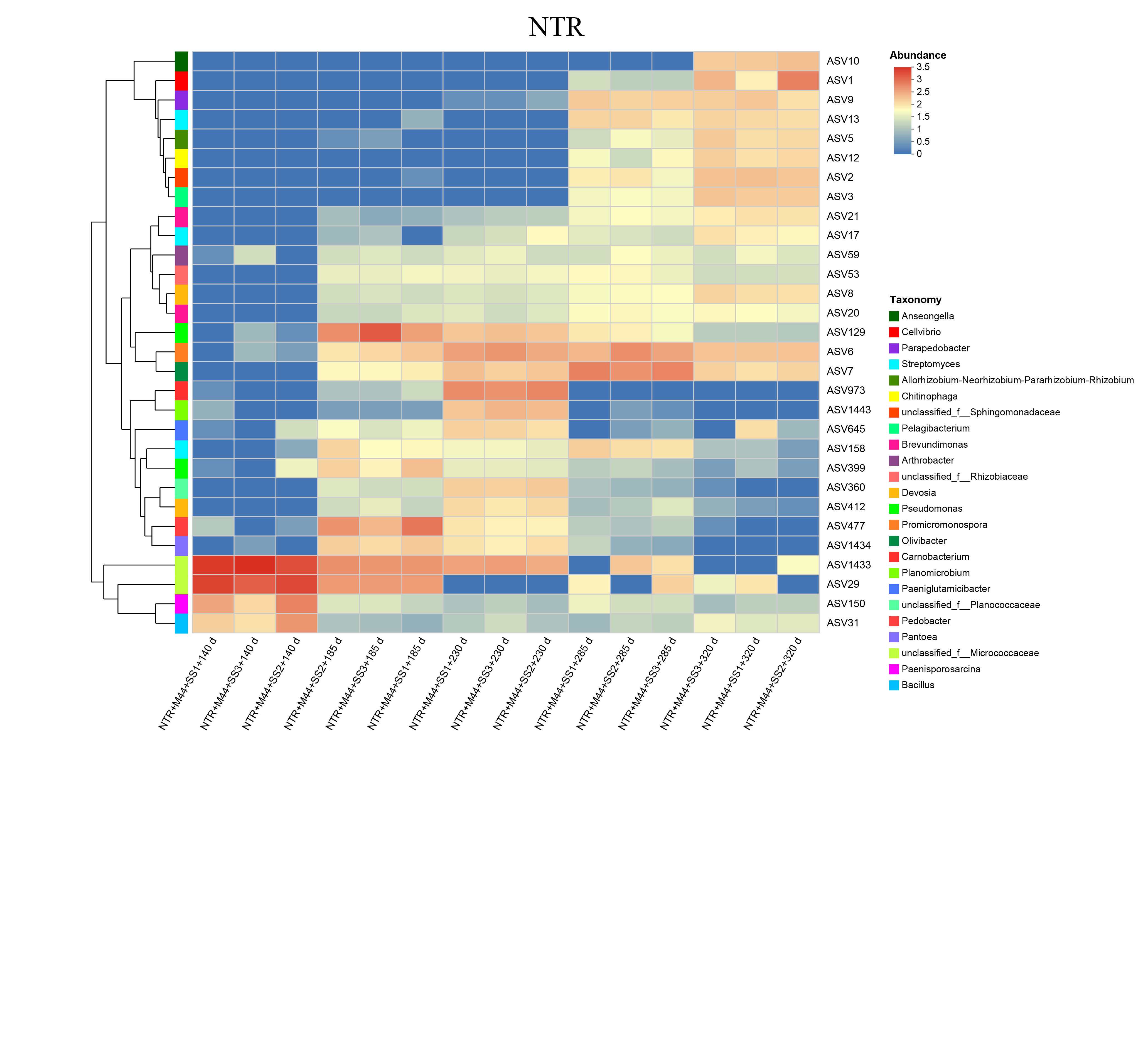


Fig S6. Analysis of species succession of soil bacteria at the genus level over degradation time under no-tillage mulched straw return with application of microbial inoculant M44.
