# Supplementary material for: The application of straw returning combined with low-temperature degrading microbial inoculant M44 in cold and arid regions promotes the efficient decomposition of returned straw through the hierarchical interaction mechanism of “key microorganisms—bacterial community structure—extracellular enzyme activity—straw degradation”
Source: Front Microbiol. 2026 Apr 29;17:1765717. doi: 10.3389/fmicb.2026.1765717 (PMC13168190; doi:10.3389/fmicb.2026.1765717)
Supplement: Supplementary file 6 [file Table_5.docx]

supplementary material

The application of straw returning combined with low-temperature degrading microbial inoculant M44 in cold and arid regions promotes the efficient decomposition of returned straw through the hierarchical interaction mechanism of "key microorganisms - bacterial community structure - extracellular enzyme activity - straw degradation"


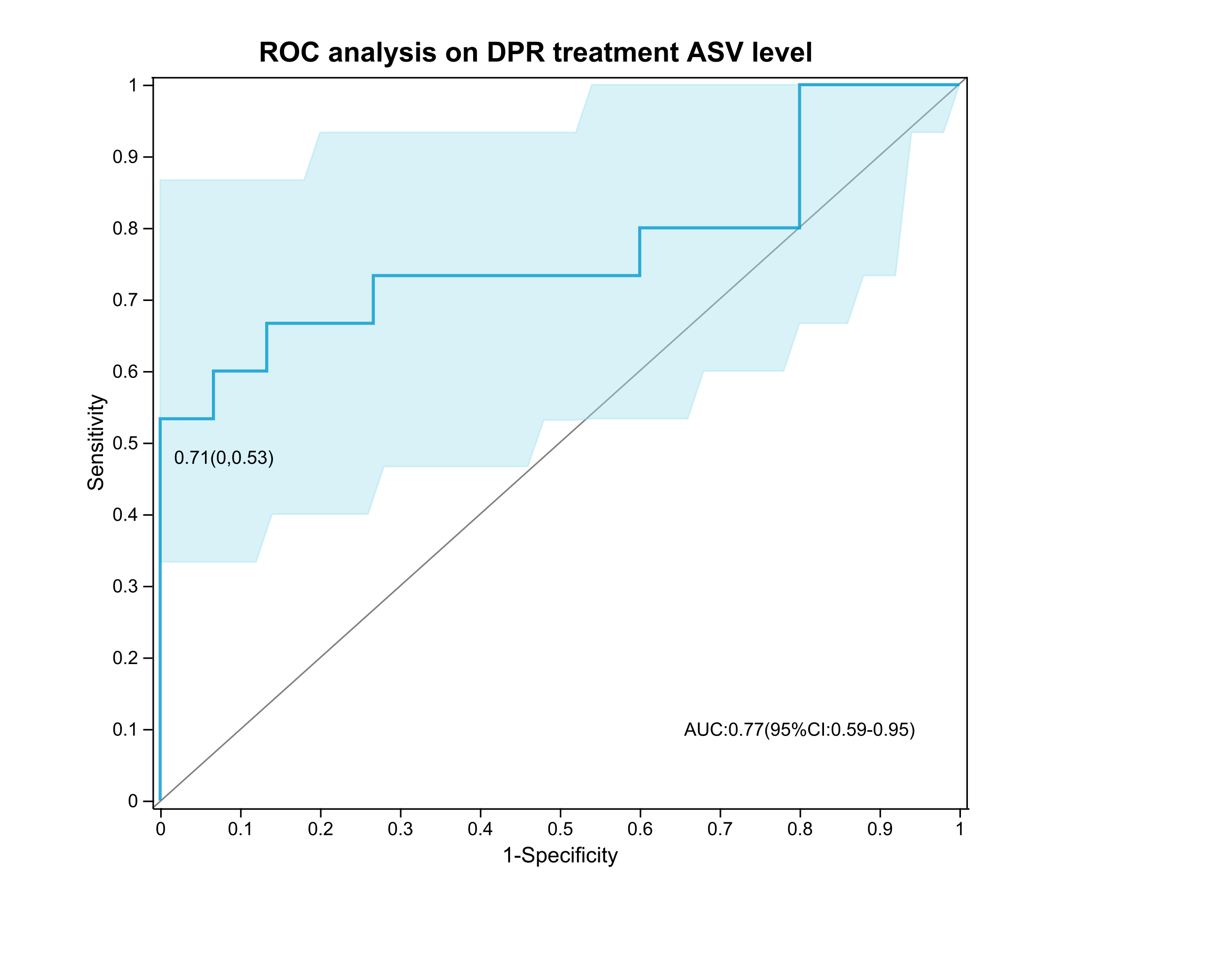


Fig S7. ROC curves of key ASVs screened from the deep tillage straw return treatment with application of microbial inoculant M44.


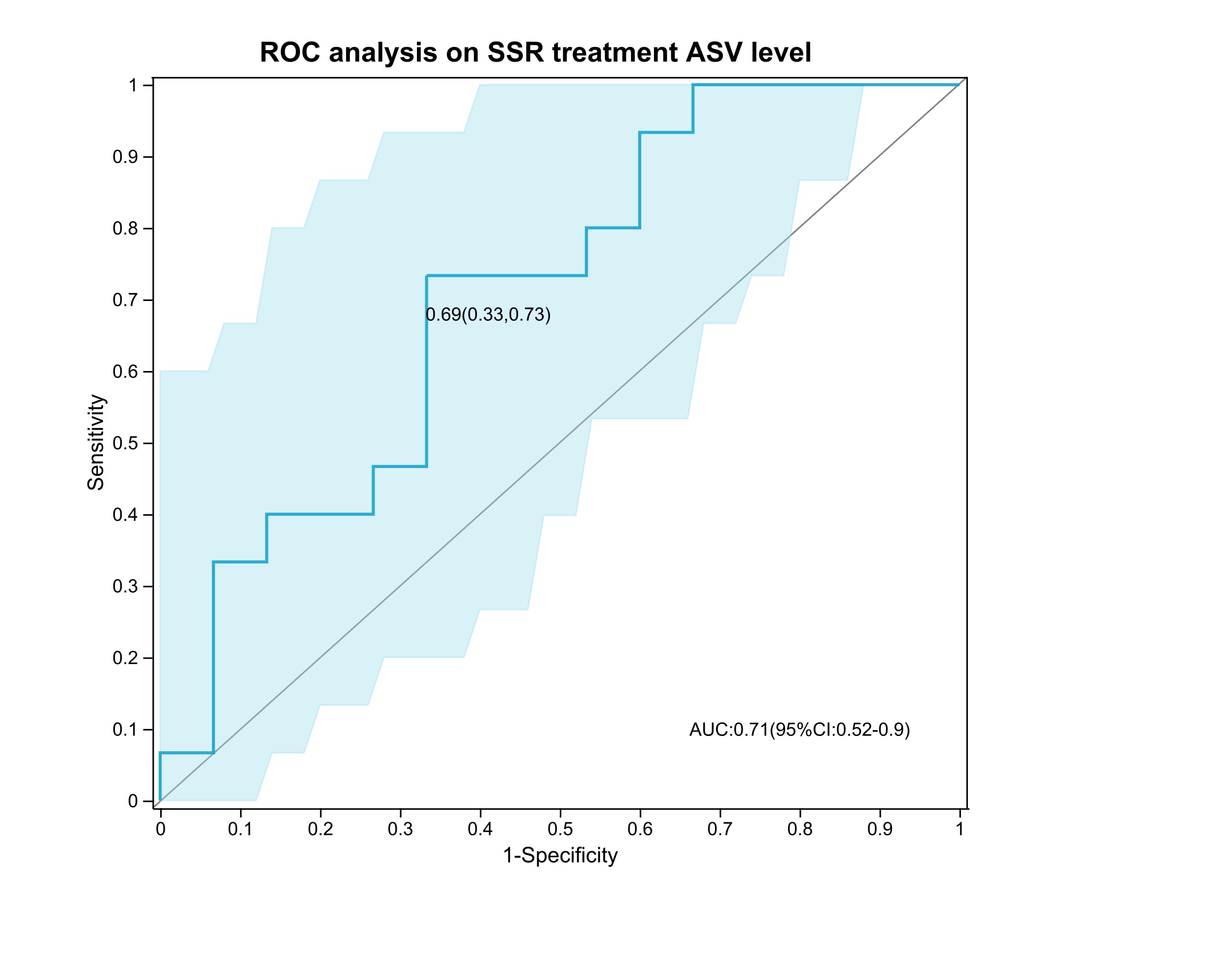


Fig S8. ROC curves of key ASVs screened from the deep subsoling mixed straw return treatment with application of microbial inoculant M44.


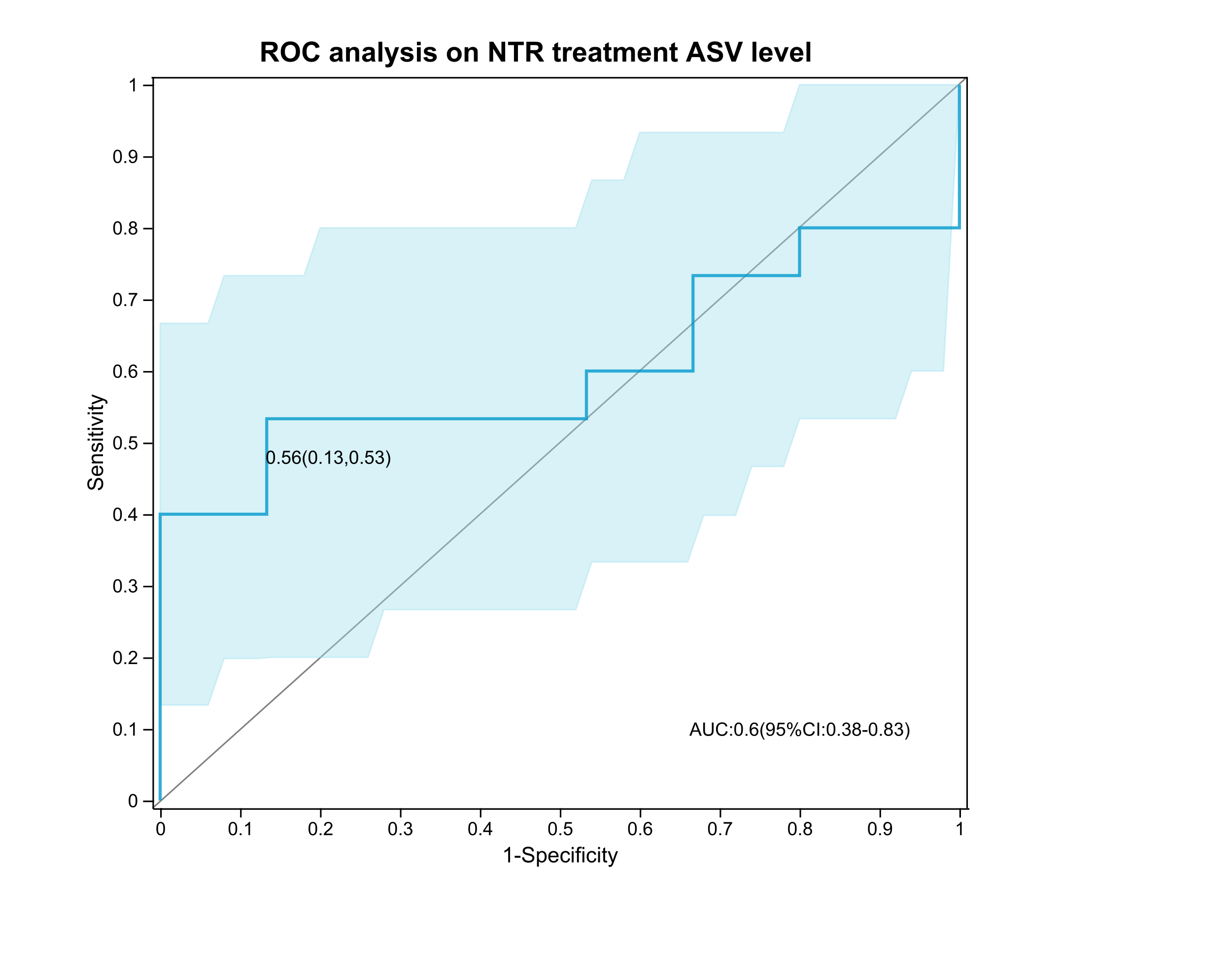


Fig S9. ROC curves of key ASVs screened from the no-tillage mulched straw return treatment with application of microbial inoculant M44.


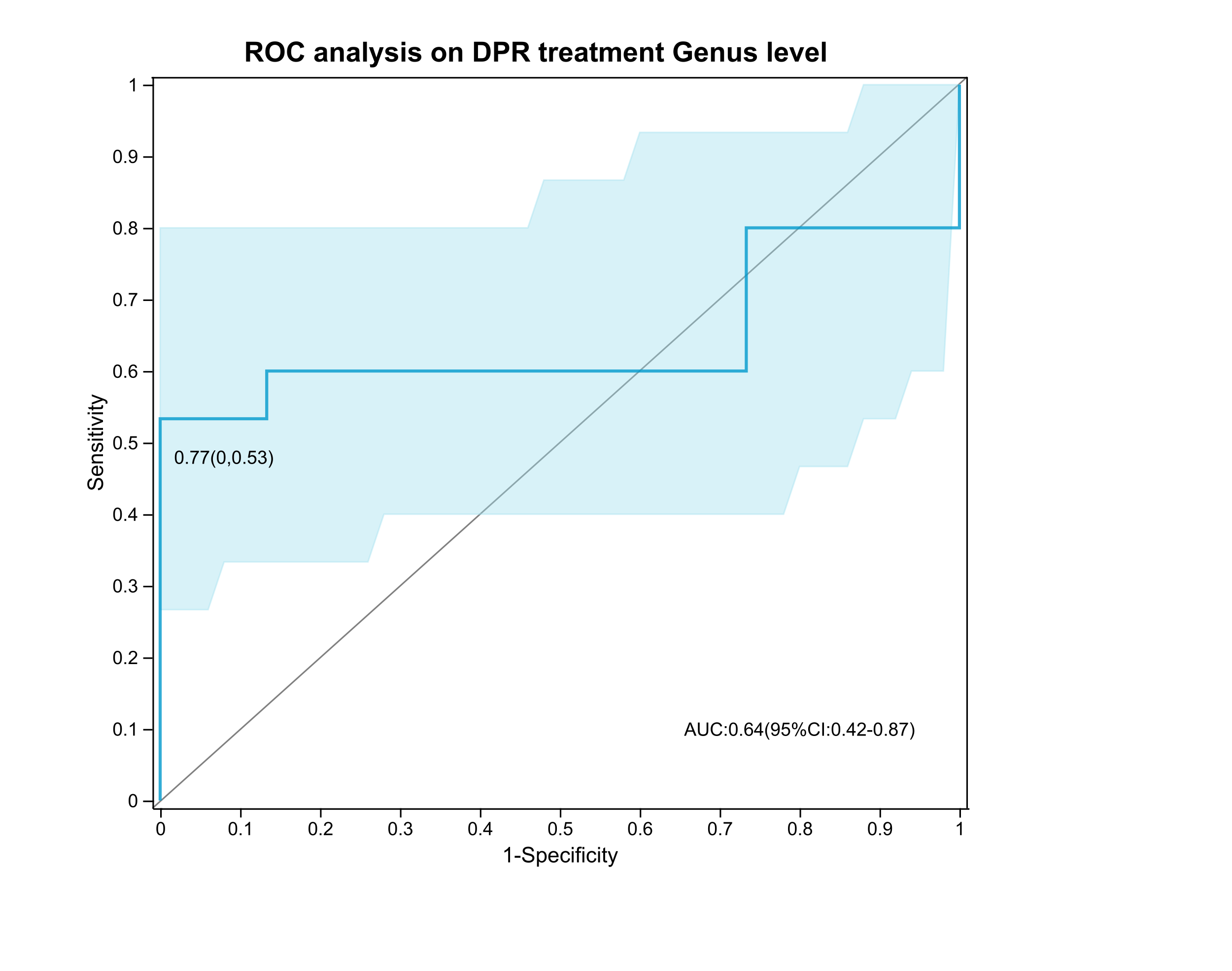


Fig S10. ROC curves of key bacterial genera screened from the deep tillage straw return treatment with application of microbial inoculant M44.


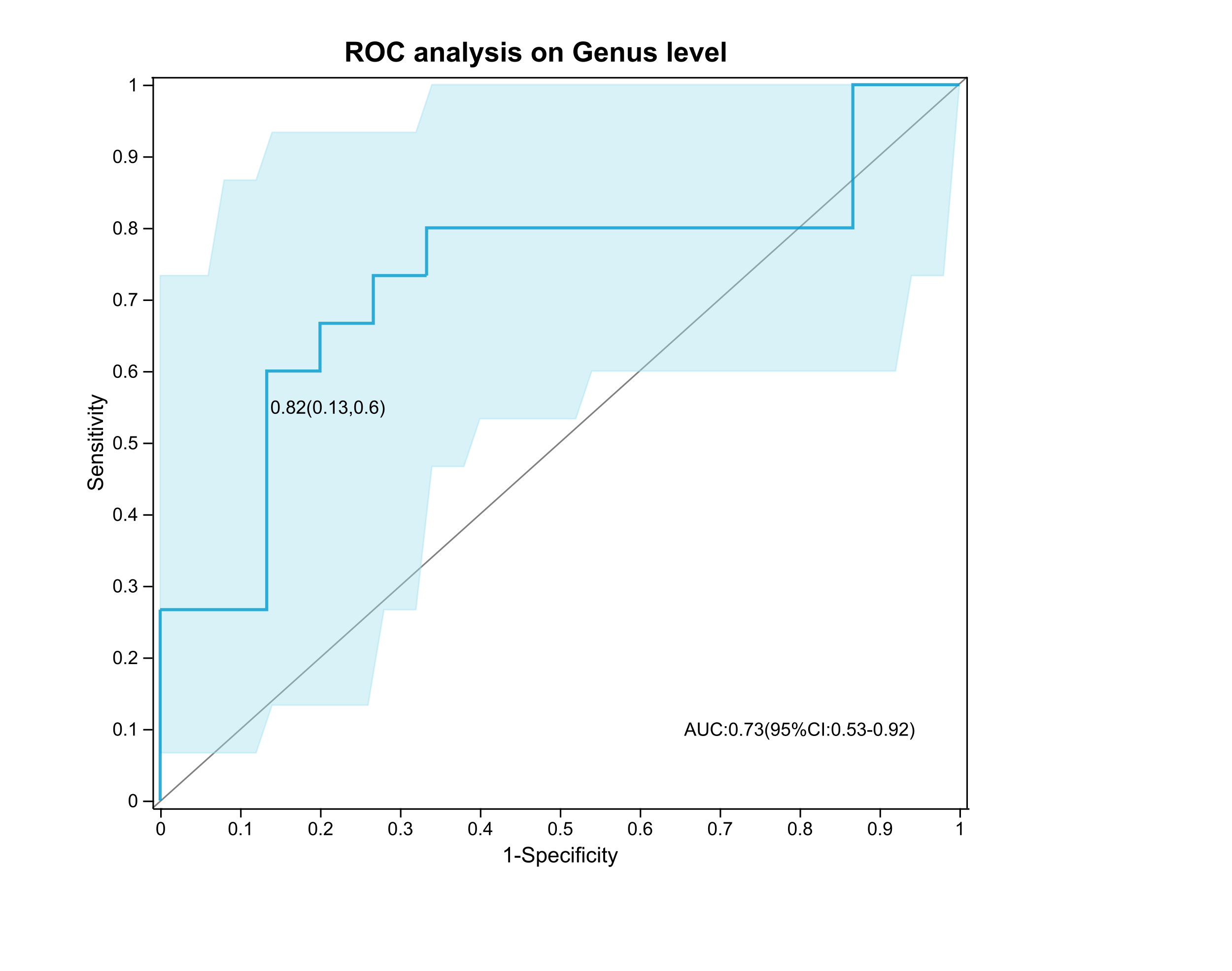


Fig S11. ROC curves of key bacterial genera screened from the deep subsoling mixed straw return treatment with application of microbial inoculant M44.


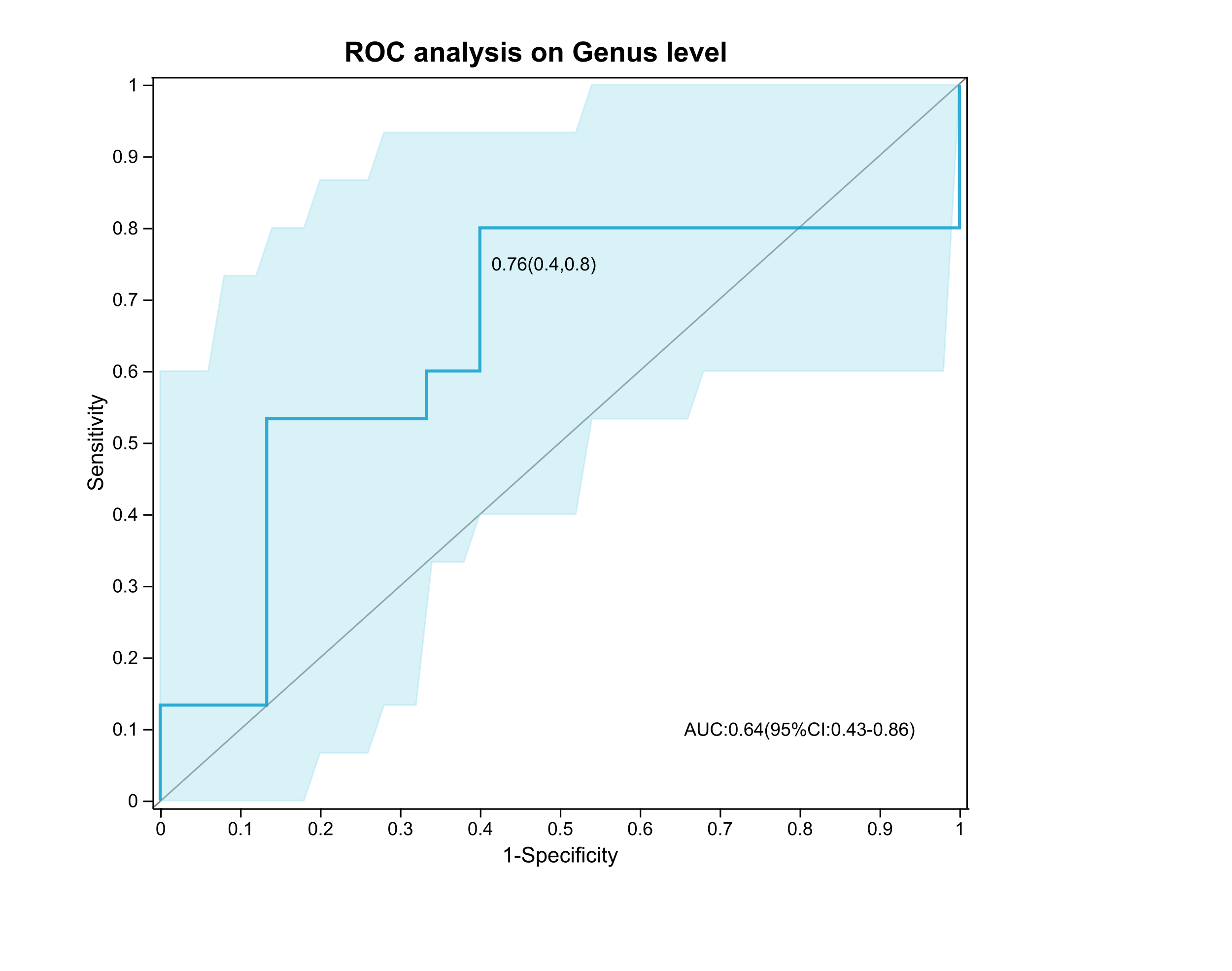


Fig S12. ROC curves of key bacterial genera screened from the no-tillage mulched straw return treatment with application of microbial inoculant M44.
