# Supplementary material for: The application of straw returning combined with low-temperature degrading microbial inoculant M44 in cold and arid regions promotes the efficient decomposition of returned straw through the hierarchical interaction mechanism of “key microorganisms—bacterial community structure—extracellular enzyme activity—straw degradation”
Source: Front Microbiol. 2026 Apr 29;17:1765717. doi: 10.3389/fmicb.2026.1765717 (PMC13168190; doi:10.3389/fmicb.2026.1765717)
Supplement: Supplementary file 7 [file Table_6.DOC]

supplementary material

The application of low-temperature degrading microbial inoculant M44 in combination with straw returning in cold and arid regions promotes the effective decomposition of returned straw through the interaction mechanism of "key microorganisms - extracellular enzyme activity - straw degradation"

Table S1 Key ASVs regulated by microbial agent M44 under different straw return methods, together with their taxonomic affiliations and functional linkages

| Return to Field Methods | Key ASVs | Taxonomic Affiliation | Enrichment Period and Correlation in Straw Degradation Process |
| --- | --- | --- | --- |
| DPR | ASV6 | Promicromonospora | Enriched across the entire straw degradation period |
|  | ASV1435 | Phyllobacterium | Enriched across the entire straw degradation period |
|  | ASV31 | Bacillus | Enriched in the early stage |
|  | ASV129 | Pseudomonas | Enriched in the early stage and significantly positively correlated with β-cellobiosidase activity |
|  | ASV477、ASV1534 | Pedobacter | Enriched in the middle stage and significantly positively correlated with β-cellobiosidase activity |
|  | ASV336 | Mycobacterium | Enriched in the middle–late stage |
|  | ASV1433、ASV1447 | unclassified_f__Micrococcaceae | Enriched in the middle–late stage |
|  | ASV1462 | Stenotrophomonas | Enriched in the middle–late stage |
|  | ASV7 | Olivibacter | Enriched in the middle–late stage and significantly positively correlated with straw degradation indicators and β-xylosidase activity |
|  | ASV1546 | Pseudoxanthomonas | Enriched in the middle–late stage and significantly positively correlated with straw degradation indicators and β-xylosidase activity |
|  | ASV1602 | Candidimonas | Enriched in the middle–late stage |
|  | ASV184、ASV412 | Devosia | Enriched in the late stage |
| SSR | ASV6 | Promicromonospora | Enriched across the entire straw degradation period |
|  | ASV29、ASV1433、ASV1548 | unclassified_f__Micrococcaceae | Enriched across the entire straw degradation period |
|  | ASV1462 | Stenotrophomonas | Enriched across the entire straw degradation period and significantly positively correlated with β-xylosidase, β-glucosidase, and leucine aminopeptidase activities |
|  | ASV158 | Streptomyces | Enriched across the entire straw degradation period and significantly positively correlated with β-xylosidase, β-glucosidase, and leucine aminopeptidase activities |
|  | ASV399、ASV1442 | Pseudomonas | Enriched across the entire straw degradation period |
|  | ASV412 | Devosia | Enriched across the entire straw degradation period |
|  | ASV477、ASV1473、ASV1534 | Pedobacter | Enriched across the entire straw degradation period |
|  | ASV1435 | Phyllobacterium | Enriched across the entire straw degradation period |
|  | ASV1546 | Pseudoxanthomonas | Enriched in a stage‑specific manner and significantly positively correlated with β-xylosidase, β-glucosidase, and leucine aminopeptidase activities |
|  | ASV12 | Chitinophaga | Enriched in a stage‑specific manner |
| NTR | ASV31 | Bacillus | Enriched in the early–middle stage |
|  | ASV150 | Paenisporosarcina | Enriched in the early–middle stage |
|  | ASV6 | Promicromonospora | Enriched in the middle–late stage and significantly positively correlated with straw degradation and β-xylosidase, β-glucosidase, and leucine aminopeptidase activities |
|  | ASV7 | Olivibacter | Enriched in the middle–late stage and significantly positively correlated with straw degradation and β-xylosidase, β-glucosidase, and leucine aminopeptidase activities |
|  | ASV129 | Pseudomonas | Enriched in the middle–late stage |
|  | ASV477 | Pedobacter | Enriched in the middle–late stage |
|  | ASV973 | Carnobacterium | Enriched in the middle–late stage |
|  | ASV1434 | Pantoea | Enriched in the middle–late stage |
|  | ASV29、ASV1433 | unclassified_f__Micrococcaceae | Enriched in a stage‑specific manner |
